# Tailoring and Identifying Brønsted Acid Sites on Metal Oxo-Clusters of Metal–Organic Frameworks for Catalytic Transformation

**DOI:** 10.1021/acscentsci.2c01140

**Published:** 2023-01-04

**Authors:** Weibin Liang, Xuelong Wang, Wenjie Yang, Shufang Zhao, Dianne Wiley, Brian S. Haynes, Yijiao Jiang, Ping Liu, Jun Huang

**Affiliations:** † School of Chemical and Biomolecular Engineering, Sydney Nano Institute, The University of Sydney, NSW2006, Australia; ‡ Chemistry Division, Brookhaven National Laboratory, Upton, New York11973, United States; § Department of Engineering, Macquarie University, Sydney, NSW2109, Australia; ∥ Department of Chemistry, Stony Brook University, Stony Brook, New York11794, United States

## Abstract

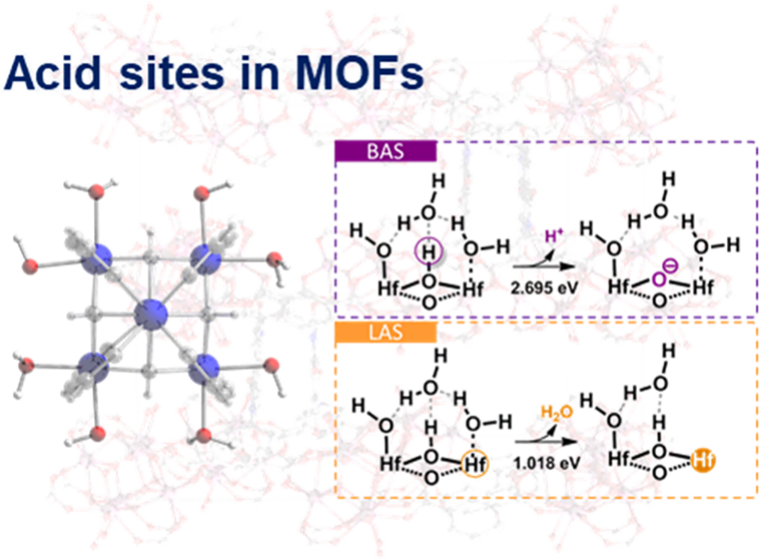

Metal–organic frameworks (MOFs) with Brønsted acidity are an alternative solid acid catalyst for many important chemical and fuel processes. However, the nature of the Brønsted acidity on the MOF’s metal cluster or center is underexplored. To design and optimize the acid strength and density in these MOFs, it is important to understand the origin of their acidity at the molecular level. In the present work, isoreticular MOFs, ZrNDI and HfNDI (NDI = *N*,*N*′-bis(5-isophthalate)naphthalenediimide), were prepared as a prototypical system to unravel and compare their Brønsted and Lewis acid sites through an array of spectroscopic, computational, and catalytic characterization techniques. With the aid of solid-state nuclear magnetic resonance and density functional calculations, Hf_6_ oxo-clusters on HfNDI are quantitatively proved to possess a higher density Brønsted acid site, while ZrNDI-based MOFs display stronger and higher-population Lewis acidity. HfNDI-based MOFs exhibit a superior catalytic performance in activating dihydroxyacetone (DHA) and converting DHA to ethyl lactate, with 71.1% selectivity at 54.7% conversion after 6 h. The turnover frequency of BAS-dominated Hf-MOF in DHA conversion is over 50 times higher than that of ZSM-5, a strong BAS-based zeolite. It is worth noting that HfNDI is reported for the first time in the literature, which is an alternative platform catalyst for biorefining and green chemistry. The present study furthermore highlights the uniqueness of Hf-based MOFs in this important biomass-to-chemical transformation.

## Abstract 2

As revealed by solid-state NMR, density functional calculations, and catalytic study, HfNDI features a higher density of acid sites and stronger Brønsted acidity than ZrNDI.

## Introduction

Over 50% of industrially important catalytic applications involve acid catalysis, such as in traditional hydrocarbon transformation and in the emerging biorefining.^1^ In comparison with liquid acid catalysts (H_2_SO_4_, H_3_PO_4_, HF, *et al.*), solid acid catalysts, such as zeolites, neat and/or modified metal oxides, functionalized silica/carbon materials, and heteropolyacids (HPAs), are more environmentally friendly in terms of corrosiveness, safety, and ease of separation/recovery.^2^ In particular, solid acid catalysts based on porous materials such as zeolites have the potential to combine the advantageous features of homogeneous catalysts (selectivity and reactivity) and heterogeneous catalysts (ease of purification and recyclability) in catalytic reactions. Metal–organic frameworks (MOFs), as a class of crystalline and porous materials, represent one of the most promising candidates in this regard. In catalysis, MOFs allow for structural refinement according to a target application at the same molecular level as homogeneous catalysts,^3^ while their high surface area and heterogeneous nature facilitate good catalytic activity and convenient purification. There are two types of acidity categorized for solid acid catalysts, namely Brønsted and Lewis acidity.^4^ MOFs have been widely reported as Lewis acid catalysts and used to catalyze many important chemical reactions such as ring opening, hydrolysis, coupling, dehydrogenation, etc.^5−7^ MOFs are constructed by coordinatively connecting inorganic metal ions or clusters with organic linkers; therefore, the coordinatively unsaturated metal clusters or open metal sites are naturally Lewis acid sites (LAS).^8^ On the other hand, Brønsted acidity has been introduced in MOFs, generally via encapsulating Brønsted acid entities within the cavities of MOFs, such as CsHSO_4_@MIL-101,^9^ H_3_PW_12_O_40_@HKUST-1 (or NENU-3a),^10^ and H_3_PW_12_O_40_@MIL-100,^11^ or dangling Brønsted acid functional groups on the organic linkers, such as −SO_3_H, −PO_3_H,^12^ and −COOH.^13^

Similar to zeotype Brønsted acid sites (BAS) originating from the bridging hydroxyl groups between silicon and aluminum atoms, the structural hydroxyl groups in the oxo-clusters of MOFs connecting two or more metal ions contribute to the Brønsted acidity. In addition, coordinating water molecules on metal sites could also generate Brønsted acidity. The presence of these hydroxyl/water-based BAS has no disruptive influence on the framework stability, and the density of acidity is not limited by the porous space. Upon dehydration/hydration, the BAS and LAS can be transferred to each other, and their ratio can be fine-tuned for a targeted reaction. However, the acid strengths of these bridging hydroxyl groups or coordinated water on MOFs are not comparable to the BAS on widely used zeolites. Improving the BAS acidity is a great challenge in MOF catalysis and attracts broad research interests from scientists and engineers.^9−17^

For acidic MOFs constructed based on metal clusters, decreasing the ligand connectivity on the metal clusters can enhance the population of the coordinatively unsaturated sites, which directly increases the acidity of MOFs. For example, the [M_6_(μ_3_-O)_4_(μ_3_-OH)_4_]^12+^ octahedron cluster supports a reduction in its connectivity to ligands from the original 12 to 10, 8, and even 6.^18^ This research explored the acidic MOFs with only four organic linkers connected to each M_6_ oxo-cluster, which reached the lowest connectivity of metal clusters and provided the maximum unsaturated sites for enhancing the acidity of the MOFs. The Zr- and Hf-MOFs are widely applied in chemical reactions as highly acidic catalysts owing to their stronger electric dipole moments compared to other metal-MOFs. Moreover, the Zr_6_ or Hf_6_ oxo-clusters like [M_6_(μ_3_-O)_4_(μ_3_-OH)_4_]^12+^ (M = Zr or Hf) keep the high stability of MOFs when decreasing the connectivity on the clusters to further enhance their acidity.

Herein, we synthesized acid MOFs by connecting Zr_6_ or Hf_6_ oxo-clusters with NDI ligands (NDI = *N*,*N*′-bis(5-isophthalate)naphthalenediimide). Both MOFs (ZrNDI (NU-1401) and HfNDI) contain eight coordinatively unsaturated sites on each M_6_ oxo-cluster, proposed for the high acidity. The Lewis/Brønsted acid properties have been characterized by ^31^P MAS NMR spectroscopies using trimethyl phosphine oxide (TMPO) as probe molecules, in combination with density functional theory (DFT) calculations. Despite similar physicochemical properties of Zr and Hf, the higher oxophilicity of Hf with respect to Zr implies a stronger Brønsted acidity on HfNDI than on ZrNDI. The ZSM-5 zeolite with dominant Brønsted acidity is applied as a reference for comparison. The dihydroxyacetone (DHA) transformation with ethanol is used as the test reaction for acid MOFs in this research since the conversion of biomass-derived DHA is an important biorefining reaction, as well as a good test reaction for acid catalysis due to its high sensitivity for the acidity features.^19,20^

## Results and Discussion

**Figure 1:**
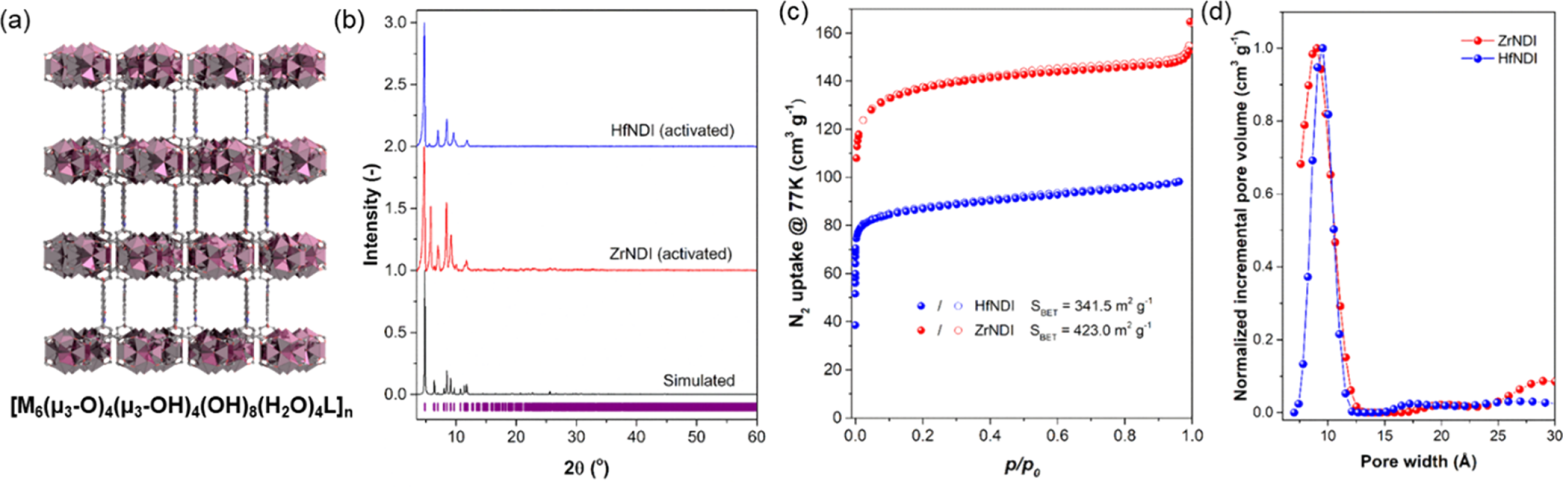
Characterization of ZrNDI and HfNDI MOFs. (a) Molecular illustration of [M_6_(μ_3_-O)_4_(μ_3_-OH)_4_(OH)_4_(H_2_O)_4_(L)]_*n*_, M = Zr for ZrNDI or Hf for HfNDI; L = NDI. (b) Simulated (black) and experimental powder X-ray diffraction patterns of activated ZrNDI (red) and HfNDI (blue). (c) N_2_ (measured at 77 K) adsorption (filled symbols)/desorption (open symbols) isotherms for ZrNDI (red) and HfNDI (blue). The calculated values of the BET surface area of the materials are shown in the legend of the figure. (d) Pore size distribution curves (calculated using the NLDFT model) for ZrNDI (red) and HfNDI (blue). The average pore sizes for ZrNDI and HfNDI were estimated at 9 Å.

The phase purity of the materials (ZrNDI and HfNDI) was characterized and confirmed by powder X-ray diffraction (PXRD). As shown in Figure 1a, the highly crystalline PXRD patterns of ZrNDI and HfNDI indicate the successful synthesis of the targeted materials. The cell parameters of ZrNDI and HfNDI were extracted from their PXRD patterns using GSAS II software^21^ and are shown in Table S1. The discrepancy between the experimental PXRD patterns of the activated ZrNDI and HfNDI and the simulated ones is attributed to the flexibility of the framework. By comparison, HfNDI processes a slightly larger unit cell than ZrNDI (Table S1). The morphology of ZrNDI and HfNDI was revealed by scanning electron microscopy (SEM) analysis and determined to be block-shape crystallites with over 10 μm in diameter (Figure S1f).

The porosity of ZrNDI and HfNDI was examined by measuring the N_2_ 77 K gas sorption isotherms. The values of the Brunauer–Emmett–Teller (BET) surface area for ZrNDI and HfNDI were determined to be 423.0 and 341.5 m^2^ g^–1^, respectively (Figure 1b). In addition, the pore size distributions calculated via nonlinear density function theory (NLDFT) show one pore feature centered at around 9 Å (Figure 1c), which is consistent with the crystallographic data (Figure S1e). The similarity of pore features in ZrNDI and HfNDI can be rationalized to the same d_0_ electronic configuration and very similar ionic radii for the Zr^4+^ and Hf^4+^ cations (79 and 78 pm, respectively).^3^

**Figure 2:**
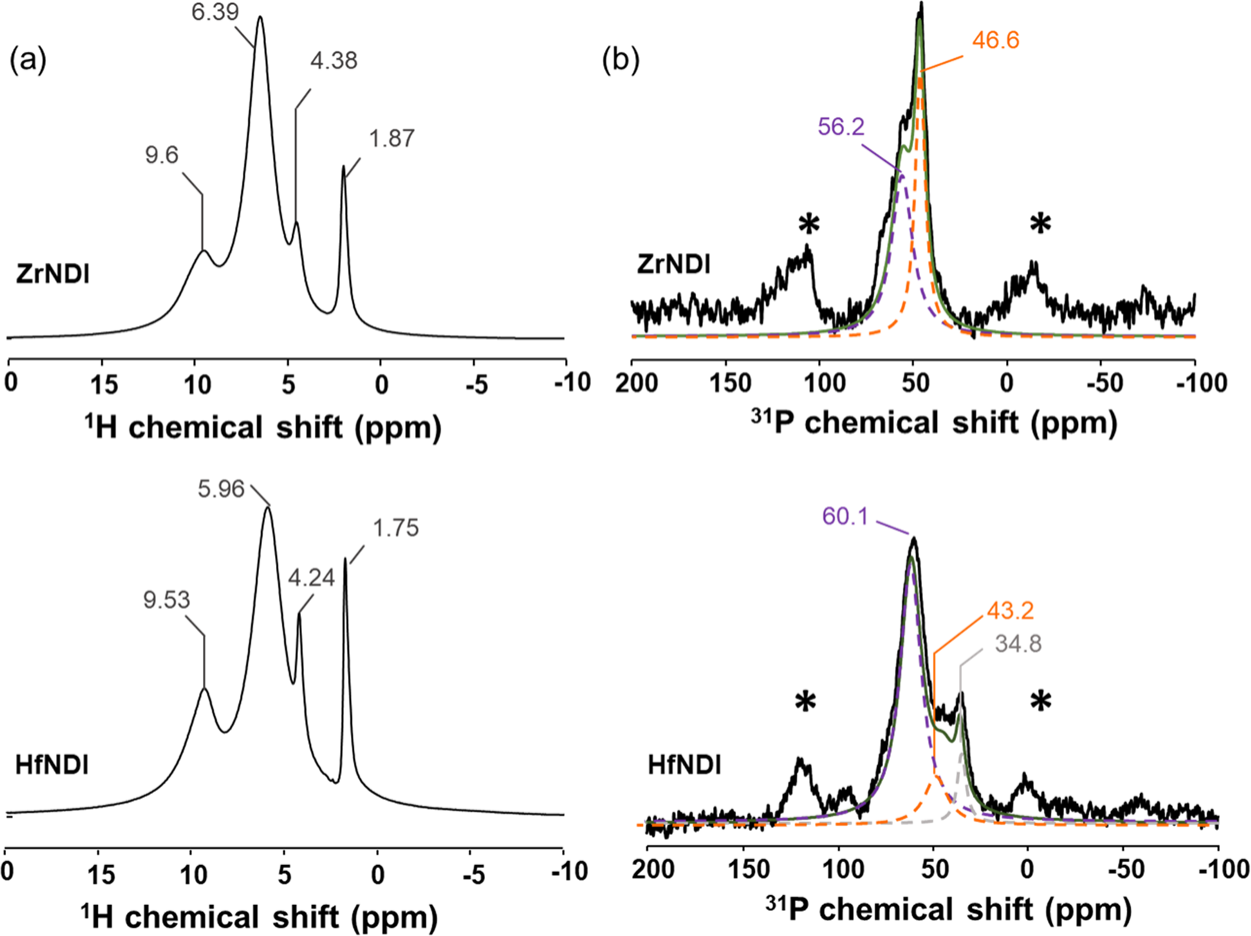
(a) ^1^H MAS ssNMR spectrum of ZrNDI and HfNDI MOFs. (b) ^31^P MAS ssNMR spectrum of ZrNDI and HfNDI MOFs. In (b), the Brønsted and Lewis acid sites in the cluster are highlighted in purple and orange colors, respectively. The ^31^P NMR signal attributed to the physiosorbed TMPO molecule is highlighted in gray color. The peaks marked with an asterisk (*) are side bands in ssNMR.

^1^H magic angle spinning (MAS) solid-state nuclear magnetic resonance (ssNMR) is used to investigate the bridging OH groups, coordinated H_2_O, and structure of ZrNDI and HfNDI. The aromatic proton of the NDI ligand can be observed at δ_1H_ ≈ 9.6 ppm (Figure 2a). The broad ^1^H signal at ∼6 ppm is assigned to the water absorbed in the materials.^22^ The ^1^H chemical shift related to the coordinated water molecules on the MOF clusters are identified at δ_1H_ = 4.38 and 4.24 ppm for ZrNDI and HfNDI, respectively.^23^ Lastly, the ^1^H signals at δ_1H_ = 1.87 ppm for ZrNDI and δ_1H_ = 1.75 ppm for HfNDI are assigned to the proton species of the coordinated hydroxyl groups in the Zr_6_ and Hf_6_ oxo-clusters, respectively.

To further explore the acidity of ZrNDI and HfNDI in-depth, we characterized the MOF materials by applying ^31^P NMR techniques with TMPO as the probe molecule. This technique has proved to be a sensitive and reliable approach to determine the acid type and strength in a solid acid catalyst.^24,25^

Figure 2b shows ^31^P MAS NMR spectra obtained for the present samples. The main signal observed at δ_31P_ = 46.6 ppm in the spectrum for ZrNDI is caused by Lewis acid sites,^4,26^ and a shoulder at δ_31P_ = 56.2 ppm relates to Brønsted acid sites.^27^ For HfNDI, three distinct peaks are observed, centered at δ_31P_ = 60.6 ppm, δ_31P_ = 43.2 ppm, and δ_31P_ = 34.8 ppm and assigned to the Brønsted and Lewis acidic species and physically adsorbed TMPO, respectively.^27,28^ By comparing the ^31^P MAS NMR spectra of ZrNDI and HfNDI, we can conclude that HfNDI possesses dominant Brønsted acid sites with a stronger acidity than ZrNDI (δ_31P_ = 60.6 ppm for HfNDI vs δ_31P_ = 56.2 ppm for ZrNDI), while ZrNDI shows a mixture of LAS/BAS and a stronger Lewis acidity than HfNDI (δ_31P_ = 46.6 ppm for ZrNDI vs δ_31P_ = 43.2 ppm for HfNDI). In fact, Hf is more oxophilic in nature than Zr, as evidenced by the higher dissociation enthalpy in typical Hf–O bonds (802 kJ mol^–1^) versus that in Zr–O bonds (776 kJ mol^–1^).^29^ Therefore, in Hf-based MOFs, the deprotonation of the coordinated −OH groups and adsorbed water molecules on the Hf_6_ oxo-clusters is expected to be more favorable, leading to a stronger Brønsted acidity. By the same principle, the weaker Zr–O bonds in Zr-MOFs promote the desorption of the coordinated water molecule on Zr_6_ oxo-clusters and expose the coordinatively unsaturated Zr site, resulting in a stronger Lewis acidity in Zr-MOFs. The composition of the Lewis/Brønsted acidity is also different for ZrNDI and HfNDI. By comparing the integrated area of the deconvoluted peaks in the ^31^P MAS NMR spectrum, the ratio between the density of Brønsted and Lewis acid sites is summarized in Table 1. In HfNDI, Brønsted acid sites are the dominant acidic species with a fraction of 83.2% in total acids, while in ZrNDI, the Lewis acid sites account for a slightly higher percentage (55.6%) than the Brønsted acid groups (44.4%). By applying ammonium dihydrogen phosphate as an external phosphorus standard, the total amounts of acid sites are calculated to be 1.56 × 10^–2^ and 2.44 × 10^–2^ mmol g^–1^ for ZrNDI and HfNDI, respectively. The density of acid sites in HfNDI is approximately 1.5 times that in ZrNDI.

**Table 1:** Acid Properties Obtained from ^31^P MAS NMR Spectroscopy Using TMPO Probe Molecules and the TOFs of ZrNDI and HfNDI MOF in the Reaction

sample	density of acid sites (mmol g^–1^)^a^	BAS (%)^b^	LAS (%)^b^	TOF (h^–1^)^c^
ZrNDI	1.56 × 10^–2^	55.63 (56.2 ppm)	44.37 (46.6 ppm)	4.5
HfNDI	2.44 × 10^–2^	83.22 (60.6 ppm)	16.78 (43.2 ppm)-	22.4

a Calculated from the quantitative evaluation of the ^31^P MAS NMR spectra (Figure 2b). Ammonium dihydrogen phosphate as an external phosphorus standard for quantitative analysis.

b BAS/LAS = Brønsted/Lewis acid site.

c TOF = turnover frequency; calculated as described in the text.

**Figure 3:**
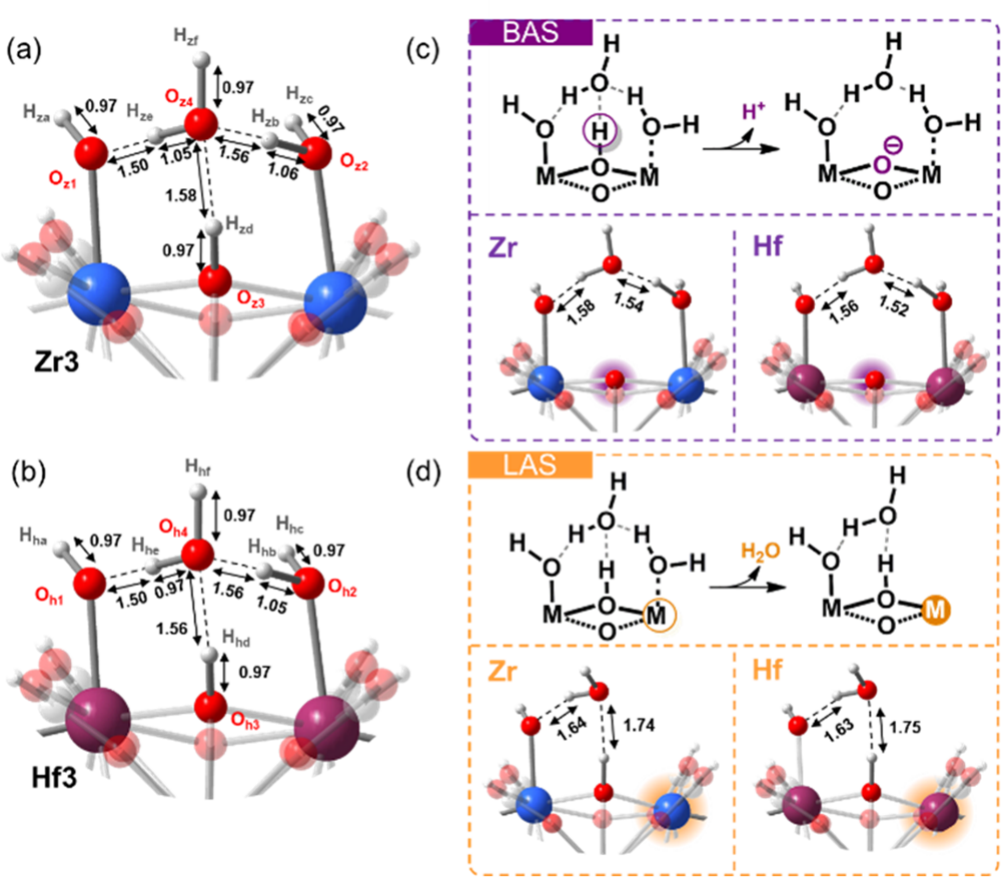
Molecular representations for the optimized proton configuration of the metal oxo-clusters in ZrNDI (a, Zr3) and HfNDI (b, Hf3). The pathway for the deprotonation of BAS is shown in the purple box (c), while the orange box (d) shows the dehydrogenation pathway for LAS.

To get a better insight into structural moieties and chemical nature of the BAS and LAS in MOF materials, DFT calculations were carried out. The coordinatively unsaturated sites in ZrNDI and HfNDI produce positive charges on the cluster, which can be compensated either by removing a positively charged proton of the μ_3_-OH groups in the oxo-clusters or, in the presence of water, by coordinating a negatively charged hydroxide to the Zr/Hf atom. However, experimental determination of the proton topology at a molecular level in the Zr- or Hf-oxo cluster is very difficult.^30^ Instead, DFT calculations proved to be a suitable tool in studying the water/hydroxyl coordinating configurations in the Zr_6_ oxo-cluster in MOFs.^23,31,32^ In the present study, different arrangements and combinations of terminal water and hydroxide on the coordinatively unsaturated sites of Zr_6_ and Hf_6_ oxo-clusters were first systematically studied and optimized using DFT methods to determine the stable proton environment in the Zr_6_ and Hf_6_ oxo-clusters (Figure S4). To simplify the DFT modeling, the coordinating ligands on the oxo-cluster in ZrNDI and HfNDI were replaced by formate as the terminal ligands. The most stable Zr_6_ or Hf_6_ oxo-cluster is coordinated by four hydroxide and four water moieties (Zr2 or Hf2 in our notation, Figure S5), where one −OH group directly attaches to one Zr or Hf atom and a water molecule is coordinated to its adjacent Zr or Hf atom. Adding an extra water molecule to Zr2 or Hf2 helps to increase the stability of the cluster by 0.560 and 0.566 eV (Zr3 or Hf3 in our notation, Figure 3 and Figure S5). The gain in stability with the extra water molecule is associated with the induced formation of hydrogen bonds with the μ_3_-OH, M–OH, and M–OH_2_ groups (M = Zr or Hf) (Figure 3).

Based on the stable metal oxo-clusters, Zr3 or Hf3 (Figure 3 and Figure S5) in ZrNDI and HfNDI, the dehydrogenation energy (DHROE) (Figure 3c) and the dehydration energy (DHRAE) (Figure 3d) were calculated using DFT to probe the acid strength of the BAS and LAS, respectively. A lower DHROE value represents a stronger BAS strength. Although both protons from μ_3_-OH and coordinating −OH/OH_2_ groups on the Zr_6_ or Hf_6_ oxo-clusters are attributed to BAS,^33^ a quantitative comparison among them regarding their acid strength is not reported yet in the literature. The DFT results in the present study show that the hydrogen from the μ_3_-OH group in the Zr_6_ or Hf_6_ oxo-cluster can be removed most easily (Figure 3and Figures S6 and S7). The μ_3_-OH groups in the Zr_6_ or Hf_6_ oxo-cluster likely serve as the strongest BAS sites of the MOF materials, while the coordinating hydroxyl group serves as the weakest (Figures S6 and S7). Energetically, the DHROE values are comparable for Zr2 versus Hf2, where the value for Hf2 is only −0.008 eV lower than that of Zr2, while the difference increases to −0.025 eV for the stable Hf3 versus Zr3, likely indicating a stronger BAS acidity in HfNDI than in ZrNDI. The opposite trends are observed when considering DHRAE. In this case, the coordinating water molecules on Zr or Hf atoms of the MOF cluster shield the Lewis acidity of the Zr or Hf atom. Thus, the DHRAE values related to the coordinating water molecules on the Zr or Hf atoms can be used to evaluate the strength of the LAS acidity in the Zr_6_ or Hf_6_ oxo-cluster. As shown in Figure 3 and Figure S8, the DHRAE values calculated for the Zr6 oxo-cluster are found to be lower than those for the Hf-based ones (Δ = −0.056 eV for Zr2 versus Hf2, Δ = −0.039 eV for Zr3 versus Hf3), and thus a stronger LAS acidity in ZrNDI than in HfNDI is expected.

These DFT results correlate well with the ssNMR measurements (*vide supra*, Figure 2 and Table 1). Both the ssNMR measurements and DFT calculations point to a stronger BAS acidity in HfNDI and a stronger LAS acidity in ZrNDI. According to the DFT calculations, the difference in behavior between Zr_6_ and Hf_6_ oxo-clusters is associated with the intrinsic property of Zr and Hf ions. According to the calculated partial density of states (pDOS, Figure S9), the Zr ion shows higher stability than the Hf ion. As compared to the Hf ion, the Zr ion destabilizes the dehydrogenated products but helps to stabilize the dehydrated product (Figure S9). Overall, the ssNMR measurements and DFT calculations unveil the optimized proton topology and provide us quantitative results to evaluate the BAS and LAS in the ZrNDI and HfNDI MOFs for the very first time in the literature.

**Figure 4:**
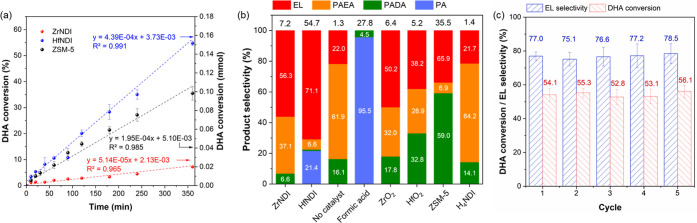
(a) Plots of the DHA conversion (in percentage) for ZrNDI (red) and HfNDI (blue) MOFs. (b) Plots of the selectivity for DHA conversion product for ZrNDI MOF, HfNDI MOF, formic acid, ZrO_2_, HfO_2_, ZSM-5, H_4_NDI ligand, and blank test (no catalyst) after 6 h of reaction in ethanol. The values of DHA conversion (in percentage) of reactions are listed in black numbers above each bar. (c) Recycling experiments of HfNDI MOF for the DHA conversion. The EL selectivity and DHA conversion of each run are presented in blue and red bars, respectively. The values of EL selectivity and DHA conversion are also listed above each bar. Error bars in the figures represent standard derivations calculated from triplicate experiments.

In the next step, we sought to examine the catalytic performance of the acid MOF catalysts in the DHA transformation reaction in ethanol at 90 °C. Due to the sensitivity of product distribution to the type and strength of acid sites, DHA transformation was chosen as a model reaction to compare the acidity features of ZrNDI and HfNDI. There are three main reactions in the catalytic DHA transformation reaction, DHA dehydration to pyruvaldehyde (PA), PA acetalization with ethanol to yield pyruvaldehyde ethyl acetal (PAEA), and PAEA isomerization to form ethyl lactate (EL).^19,20^ To achieve high EL selectivity, it is thus vital to match the reaction rate of the three reactions by optimizing the features and content of the acid sites. In the present study, it is noteworthy that HfNDI outperformed ZrNDI after a 6 h reaction in terms of both DHA conversion (54.7% versus 7.2%, Figure 4a and 4b) and EL selectivity (71.1% versus 56.3%, Figure 4b and Figure S12). The kinetic data (Figure 4a) show the initial reaction rate to be approximately 8 times greater for HfNDI (8.78 μmol min^–1^ g^–1^) than for ZrNDI (1.03 μmol min^–1^ g^–1^). The corresponding initial turnover frequencies (TOF, based on the total amount of acid sites (*vide supra*), Table 1) were 22.4 and 4.5 h^–1^ for HfNDI and ZrNDI, respectively. Given the similarity of pore features in these two materials (as evidenced by N_2_ 77 K sorption analysis and PXRD measurements, Figure 1), we conclude that their very different performances in the DHA transformation (Figure 4) is attributable to their differing acidities.

To elucidate the advantages of MOF acid catalyst, conversion of DHA in ethanol was also performed using various reference materials under the same reaction conditions and mass of catalyst. In the absence of acid catalyst, only 1.3% DHA was converted, mainly to PAEA (61.9% selectivity). Formic acid (FA, p*K*_a_ = 3.75), a strong liquid Brønsted acid, produced 27.8% DHA conversion with 95.5% selectivity to PA (Figure 4b), consistent with the literature result showing that Brønsted acids can efficiently catalyze DHA-to-PA conversion.^34^ A similar experimental observation was reported for Nafion, which contains sulfonic acid; in the absence of LAS, no EL production was detected,^27^ even though Nafion efficiently catalyzes DHA-to-PA conversion. Experiments using FA as a Brønsted acid catalyst and PA as a reaction precursor further confirm FA’s inefficiency in catalyzing PA to EL via PAEA or PADA (Figure S10). After 6 h of reaction, only 4% PA conversion with a broad product distribution was detected in the case of FA, which is not significantly different from the catalytic results observed in the absence of catalyst (Figure S10). A high PA conversion with a high EL selectivity was observed in the presence of ZrNDI or HfNDI MOFs (Figure S10). A slightly higher PA conversion was observed for HfNDI, as compared to ZrNDI, which is attributed to its higher density and strength of Brønsted acid sites (Figures 2 and 3, Table 1). Interestingly, ZrNDI is a better catalyst for PA transformation than it is for DHA under analogous reaction conditions (52.6% PA conversion versus 7.2% DHA conversion, Figure 4 and Figure S10). Considering that such a huge improvement in substrate conversion is not observed in the case of HfNDI (67.4% PA conversion versus 54.7% DHA conversion, Figure 4 and Figure S10), we proposed that the low DHA conversion observed in the case of ZrNDI is mainly the result of its low Brønsted acidity, which is apparently insufficient to drive the DHA-to-PA dehydration reaction efficiently. These control experiments highlighted the importance of the coexistence of Brønsted/Lewis acid sites in HfNDI in catalyzing an effective and efficient DHA-to-EL transformation reaction. Moreover, the H_4_NDI ligand was found to be inactive, yielding only 1.4% DHA conversion. These experimental results confirm that the superior catalytic activity for HfNDI does not originate from residual formate and/or H_4_NDI ligand within the MOF materials. BAS in HfNDI is the main driver for the DHA conversion as confirmed by *in situ*^1^H NMR in the Supporting Information as shown in Figures S13–S16.

To further demonstrate the essential role of the Zr_6_ and Hf_6_ oxo-clusters in the reaction, their oxide counterparts, ZrO_2_ and HfO_2_, were tested as catalysts. In fact, these oxides are reported to be active in DHA conversion at elevated temperature (>130 °C).^35,36^ Brei et al. reported ZrO_2_ to catalyze the DHA transformation in ethanol at 130–140 °C, yielding ∼60% conversion with a broad product distribution (only 7% selectivity to EL).^35^ In another publication, ZrO_2_ and HfO_2_ were reported to catalyze cellulose transformation into lactic acid (∼20% selectivity) in H_2_O at 200 °C.^36^ Under our reaction conditions, only 6.4% and 5.2% DHA conversion was observed for ZrO_2_ and HfO_2_, respectively, with poor product selectivity (Figure 4b). This comparison unambiguously confirms the essential role of the coordinatively unsaturated sites and the charged/coordinatively compensating −OH/H_2_O groups on the Zr_6_ and Hf_6_ oxo-clusters in generating the highly active acid sites in MOFs. Lastly, an aluminum-doped zeolite, ZSM-5, was used as a heterogeneous catalyst for DHA conversion. The ZSM-5 was prepared in our lab according to the published method.^37^ After 6 h of reaction at 90 °C, 35.5% DHA conversion was achieved with a broad product distribution: 59.0%, 65.9%, and 6.9% as PADA, EL, and PAEA, respectively. The broad product distribution dominated by PADA can be rationalized by the presence of strong Brønsted acid sites in the ZSM-5 catalyst.,^38,39^ illustrating the advantage of moderate Brønsted acidity with Lewis acid sites in HfNDI in catalyzing a selective DHA-to-EL transformation reaction. The density of acid sites in ZSM-5 is 0.82 mmol g^–1^.^40^ Thus, the TOF for ZSM-5 is calculated to be 0.43 h^–1^, which is much lower as compared to HfNDI.

Encouraged by the superior performance of HfNDI toward DHA transformation, we sought to examine the recyclability of the acid catalyst, an important performance factor for practical applications. We successfully reused HfNDI at least five times without significant reduction in reaction activity and product selectivity (Figure 4c). Importantly, PXRD measurements confirmed the integrity of the MOF catalyst after catalytic reactions (Figure S11).

The advantage of using Hf_6_ oxo-clusters in the DHA-to-EL transformation reaction was further tested and confirmed using another two Hf-based MOFs, Hf-STA-26 and Hf-MOF-808 (Figures S17–S20). Similar to HfNDI, both Hf-STA-26 and Hf-MOF-808 possess intrinsic coordinatively unsaturated sites for acid catalysis. A noteworthy result is that Hf-STA-26 can catalyze a complete DHA conversion with a 84.1% EL selectivity within 6 h (Figure S18). We attributed the superior catalytic performance in Hf-STA-26 to its more open pore structure (Figures S16, S17, and S19) as compared to HfNDI.

## Conclusions

In this work, two isoreticular MOFs, ZrNDI and HfNDI, with the same coordinate environment and similar pore geometry were prepared. The coordinatively unsaturated sites on the metal cluster and their charged/coordinative compensating groups endow these MOFs with Brønsted/Lewis acidity. The acidity in ZrNDI and HfNDI was probed by spectroscopic and computational characterization techniques. As evidenced by ssNMR spectroscopy, these two isoreticular MOF acids featured different BAS/LAS strengths and densities. HfNDI features a higher density of acid sites and stronger Brønsted acidity as compared to ZrNDI. Particular attention has been paid to the hydration state of the active sites on the materials and thus the origin of their Brønsted/Lewis acidity. Modeling by DFT shows that, in the most stable proton configuration, one hydroxyl group is chemisorbed on a Zr or Hf atom with one physisorbed water molecule on its adjacent one, while another water molecule is stabilized on the cluster via hydrogen bonding interactions. The strongest Brønsted acid site is shown to originate from the μ_3_-OH groups on the SBUs (SBU = secondary building unit). The stronger Brønsted acidity in HfNDI is evidenced by a lower dehydrogenation energy as compared to ZrNDI, while the stronger Lewis acidity in ZrNDI is evidenced by a lower dehydration energy. The DFT calculations correlate closely with the ssNMR results. Such a Brønsted/Lewis acid site construct may be quite generalizable in Zr- and Hf-type MOFs.

Our results have provided, for the first time, a qualitative and quantitative study to directly compare the nature of acidity in isoreticular Zr- and Hf-MOFs. Dihydroxyacetone (DHA) transformation reaction was chosen as a model reaction to test and compare the catalytic performance of ZrNDI and HfNDI. HfNDI outperforms ZrNDI in both reaction kinetics and chemoselectivity, with a quantitative DHA conversion (54.7%) and a 71.1% ethyl lactate yield. Combining the spectroscopic data, DFT calculations, and catalytic reaction results, we rationalized that the superior DHA-to-EL catalytic performance of HfNDI (versus ZrNDI) is attributed to its dominant Brønsted acid sites and the fact that its TOF is much higher than that of ZSM-5 zeolite. Our results have demonstrated the unique attributes of Hf-MOFs featured by superior stability and Brønsted acidity that allow them to be applied as heterogeneous catalysts in biobased chemical synthesis. The present work can also serve as a prototype to demonstrate how a MOF can be designed and fine-tuned to satisfy the requirements for the creation of an effective and efficient acidity.

## Experimental Section

All chemicals and solvents were purchased from commercial sources and used as received without further purification.

### Syntheses

#### Ligand Synthesis

*N*,*N*′-Bis(5-isophthalic acid)naphthalenediimide ligand (H_4_NDI) and 5′-(4-carboxyphenyl)-2′,4′,6′-trimethyl-[1,1′:3′,1″-terphenyl]-4,4″-dicarboxylic acid (H_3_CTTA) were synthesized according to the literature.^41,42^

### MOF Synthesis

ZrNDI was synthesized solvothermally as described in the literature with slight modification.^40^ In a typical procedure, 14.4 mg of ZrCl_4_, 6.6 mg of H_4_NDI, 2 mL of formic acid, 3.6 mL of DMF, and 40 μL of H_2_O were mixed and sealed in a 25 mL glass vial. Thereafter, the mixture was heated in a preheated oven at 130 °C for 48 h. The resulting ZrNDI samples were collected via centrifugation and washed sufficiently with DMF (3 × 5 mL) over a period of 24 h to remove the unreacted metal and ligand precursors. Thereafter, ZrNDI (∼100 mg) was soaked in an HCl/DMSO mixture (10 mL of DMSO and 0.6 mL of 8 M aqueous HCl) at 100 °C for 16 h. After cooling to room temperature, the MOF solids were isolated by centrifugation and solvent-exchanged with ethanol. To an ethanol suspension of ZrNDI (∼10 mg mL^–1^), triethylamine (TEA, 30 μL) was added. The mixture was stored at room temperature for another 16 h. The resulting ZrNDI samples were then Soxhlet washed with ethanol overnight. The materials were then collected and fully activated at 80 °C under dynamic vacuum for 12 h to afford activated ZrNDI. The synthesis protocol of HfNDI was analogous to that of ZrNDI with the use of HfCl_4_ (20 mg) instead of ZrCl_4_ (14.4 mg).

Hf-MOF-808^43^ and Hf-STA-26^44^ were prepared according to the published protocol using HfCl_4_ as a metal precursor and formic acid as a modulating agent. The postsynthesis washing protocol for Hf-MOF-808 and Hf-STA-26 was analogous to that of ZrNDI and HfNDI.

### Characterization

#### N_2_ 77 K Sorption Analysis

N_2_ sorption isotherms were measured on an autosorb iQ instrument (Quantachrome INSTRUMENTS Inc.). Approximately 50 mg of the powdered solid was loaded into a glass analysis tube and outgassed under dynamic vacuum at 80 °C for 12 h. N_2_ adsorption and desorption isotherms were measured at 77 K, and data was analyzed using Brunauer–Emmett–Teller (BET) analysis model^41^ to determine the surface area. The pore size distribution calculations were carried out using the ASiQwin software DFT package (Quantachrome INSTRUMENTS Inc.). Prior to analysis, the as-synthesized materials were washed with dilute HCl in dimethyl sulfoxide (DMSO), followed by Soxhlet exchange with ethanol for 12 h. Thereafter, ZrNDI or HfNDI were activated at 80 °C under dynamic vacuum for 12 h.

#### Powder X-ray Diffraction (PXRD)

PXRD measurements were performed on a PANalytical XPert Pro powder diffractometer fitted with a solid-state PIXcel detector (45 kV, 40 mA, 1° divergence and antiscatter slits, and 0.3 mm receiver and detector slits) using Cu–Kα (λ = 1.5406 Å) radiation.

#### Attenuated Total Reflection Fourier Transform Infrared Spectroscopy (ATR-FTIR)

The ATR-FTIR measurements were conducted on a Thermo Nicolet iS50 infrared spectrometer.

#### Scanning Electron Microscopy Analysis (SEM)

The SEM images were recorded using a JEOL 2200FS instrument.

#### Catalytic DHA Transformation Reaction

In a typical catalytic run, 50 mg of the MOF solid acid catalyst was loaded into a pressure vessel (Chemglass, 15 mL HW Pressure Vessel), followed by the addition of 1.5 mL of 0.2 M DHA (in ethanol with nonane (1 μL mL^–1^) as the internal standard). Thereafter, the reaction vessel was tightly sealed and incubated in a preheated oil batch under vigorous stirring at 90 °C for 6 h. For kinetic experiments, the reaction mixture (0.06 mL) was sampled at regular time intervals (0, 10, 20, 30, 40, 60, 90, 120, 180, 240, and 360 min). After centrifugation, the supernatant (40 μL) of the sampled reaction solution was mixed with 960 μL of ethanol prior to GC measurements. The catalytic reaction products were determined and quantified on a Shimadzu GCMS-QP2010 Ultra GC. The GC was equipped with a RTX-5 capillary column (25 m × 0.32 mm × 5 μm) and an FID detector. DHA conversion was calculated to be the ratio between the total molar amount of catalytic products (PA, PAEA, PADA, and EL) and the initial molar amount of DHA. Product yields were calculated with the internal standard taking into account the respective sensitivity factors. The sensitivity factors of PA, PAEA, PADA, and EL were calculated based on the reported method.^45^ Values of product selectivity were determined to be the ratio between the molar amount of each catalytic product (PA, PAEA, PADA, or EL) to the total molar amount of all catalytic products (PA, PAEA, PADA, and EL).

The TOF value was calculated by using the following equation: TOF = *ṅ*_reactant_/*n*_catalyst_, where *ṅ*_reactant_ represents the hourly molar rate of reactant conversion (calculated based on Figure 4) and *n*_catalyst_ is the moles of acid sites in the catalyst applied in the reaction. *n*_catalyst_ = *m*_catalyst_ × *D*_acid groups_, where *m*_catalyst_ is the weight of the catalyst employed in the reaction (50 mg) and *D*_acid groups_ is the specific molar acid site density (mmol g^–1^) in the MOF catalyst calculated from the ^31^P MAS NMR spectrum.

#### Recycling Catalytic Activity of HfNDI

Recycling tests were performed to examine the stability of the MOF acid catalysts. After the DHA conversion reaction (90 °C, 6 h), the supernatant of the reaction mixture was removed via centrifugation and examined by GC analysis. The residual catalyst was then washed five times with successive steps of the addition of 2 mL of ethanol, centrifugation, and the removal of the liquid. The MOF powder was dried overnight in a preheated oven at 343 K. Thereafter, fresh DHA alcoholic solution (0.2 M) was mixed with the recovered MOF catalyst to commence the next reaction cycle at 90 °C.

#### Solid-State Magic Angle Spinning NMR Experiments

One-dimensional (1D) ^1^H and ^31^P MAS NMR spectra were recorded at 11.7 T on a Bruker Avance III 500 MHz spectrometer at resonance frequencies of 500.1 and 202.5 MHz, respectively. Prior to measurements, MOF samples were loaded in a rotor and spun at 12 kHz. Single-pulse excitation (90°) with recycling delays of 5 and 2 s was applied for ^1^H and ^31^P MAS NMR measurements, respectively.

Prior to TMPO loading, the MOF samples were heated at 433 K overnight under dynamic vacuum to facilitate dehydration. The dehydrated sample and an adequate amount of TMPO were then filled into a homemade rotor. To eliminate the physiosorbed TMPO molecules and enable homogeneous TMPO loading, the homemade rotor was then put into a glass tube and heated at 433 K under dynamic vacuum for 2 h. Thereafter, the TMPO-loaded MOF samples were loaded and sealed into MAS rotors. The rotors were then subjected to ^31^P MAS NMR experiments. All operations were conducted in an Ar-environment glovebox or using Schlenk line techniques.

#### Density Functional Theory Calculations

DFT calculations were performed with the Vienna Ab Initio Simulation Package (VASP), using the projection-augmented wave (PAW) approach. The generalized gradient approximation (GGA) type exchange-correlation functional in the parametrization of Perdew, Burke, and Ernzerhof (PBE) was adopted. The energy cutoff for the basis set of wave functions was set to 520 eV. To simplify the DFT modeling, the coordinating ligands on the oxo-cluster in ZrNDI and HfNDI were replaced by formate as the terminal ligands in the unit cell with lattice parameters determined by PXRD. Atomic configurations containing different numbers of terminal water and hydroxide in all possible inequivalent arrangements on the coordinatively unsaturated sites of Zr_6_ and Hf_6_ oxo-clusters were built and used for structural optimization. Due to the rather large unit cell size, only the gamma point of the Brillouin zone was used for the integration in reciprocal space. For the relaxation of the clusters, the forces felt by each atom were well converged to a level smaller than 0.01 eV/Å. After obtaining the ground-state configuration of the Zr_6_/Hf_6_ oxo-cluster, the dehydrogenation energy (DHROE) was calculated as
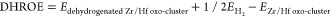
where *E* represents the total energy of dehydrogenated oxo-clusters, gas phase hydrogen, and pristine oxo-cluster, respectively. The dehydration energy (DHRAE) was calculated as
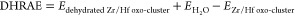
where *E* represents the total energy of dehydrated oxo-clusters, gas phase water, and pristine oxo-cluster, respectively.

## Supporting Information Available

Structural description and characterization of ZrNDI and HfNDI; solid-state nuclear magnetic resonance characterization of ZrNDI and HfNDI; density functional theory calculations; catalytic performance of ZrNDI and HfNDI in the DHA transformation reaction; solution-state NMR experiment; catalytic DHA-to-EL transformation reaction using Hf-MOF-808 and Hf-STA-26 (PDF)

## Dedication

Dedicated to Professor Jianzhong Chen on the occasion of his 70th birthday.
