# Supplementary material for: Tailoring and Identifying Brønsted Acid Sites on Metal Oxo-Clusters of Metal–Organic Frameworks for Catalytic Transformation
Source: ACS Cent Sci. 2023 Jan 4;9(1):27–35. doi: 10.1021/acscentsci.2c01140 (PMC9881200; doi:10.1021/acscentsci.2c01140)
Supplement: Supplementary file 1 — oc2c01140_si_001.pdf [file oc2c01140_si_001.pdf]

Supporting information for

Tailoring and Identifying Brønsted Acid Sites on Metal Oxo-cluster of Metal-Organic Framework for Catalytic Transformation

Weibin Liang,[a]† Xuelong Wang,[b]† Wenjie Yang,[a] Shufang Zhao,[a] Dianne Wiley,[a] Brian S. Haynes,[a] Yijiao Jiang,[c] Ping Liu,[b,d]* Jun Huang[a]*

Dedication †These authors contribute equally.

[a] Dr. W. Liang, Mr. W. Yang, Dr. S. Zhao, Prof. D. Wiley, Prof. B. S. Haynes, Prof. J. Huang
School of Chemical and Biomolecular Engineering, Sydney Nano Institute, the University of Sydney, NSW 2006, Australia
[b] Dr. X. Wang, Prof. P. Liu
Chemistry Division, Brookhaven National Laboratory, Upton, NY 11973, USA
[c] A/Prof. Y. Jiang
Department of Engineering, Macquarie University, Sydney, NSW 2109, Australia
[d] Prof. P. Liu
Department of Chemistry, Stony Brook University, Stony Brook, Stony Brook, NY 11794, USA
*Corresponding authors: Email: jun.huang@sydney.edu.au; pingliu3@bnl.gov

Table of content

Content		Page
S1.	Structural description and characterization of ZrNDI and HfNDI	S2
S2.	Solid state nuclear magnetic resonance characterization of ZrNDI and HfNDI	S4
S3	Density functional theory calculations	S5
S4.	Catalytic performance of ZrNDI and HfNDI in DHA transformation reaction	S9
S5.	Solution state NMR experiment	S10
S6	Catalytic DHA-to-EL transformation reaction using Hf-MOF-808 and Hf-STA-26	S14
S7.	References	S18

S1. Structural description and characterization of ZrNDI and HfNDI

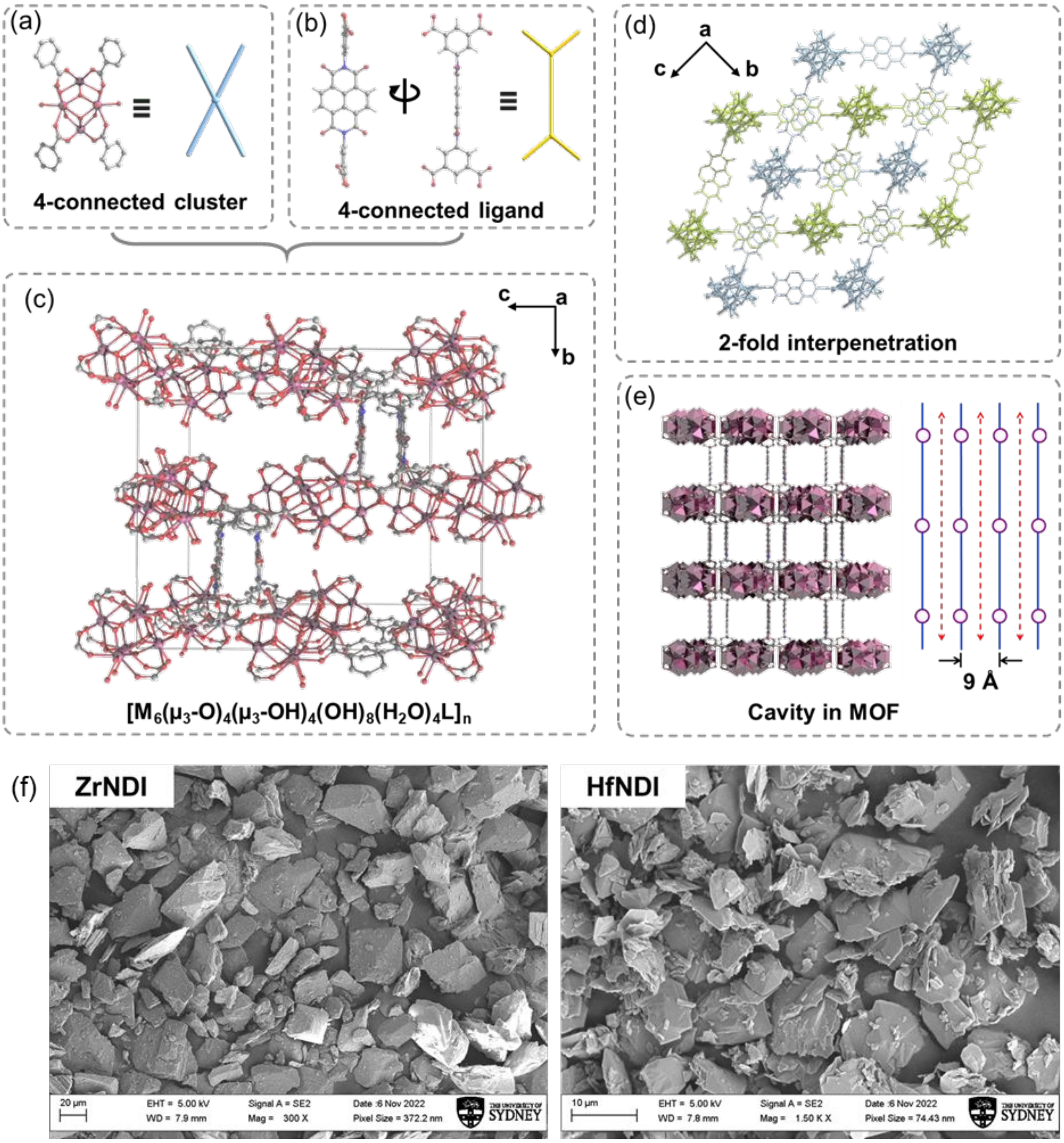

Figure S1. Structural description of ZrNDI and HfNDI. (a) a four-connected cluster in MOF; (b) four-connected N,N'-bis(5-isophthalic acid)naphthalenediimide ligand (H_4NDI); (c) unit cell of $[M_6(\mu_3\text{-}O)_4(\mu_3\text{-}OH)_4(OH)_4(H_2O)_4(NDI)]_n$, M = Zr for ZrNDI or Hf for HfNDI); (d) The arrangement of the molecular building blocks in ZrNDI and HfNDI. The interpenetrated nets are indicated in different colors; (f) SEM images of ZrNDI and HfNDI.

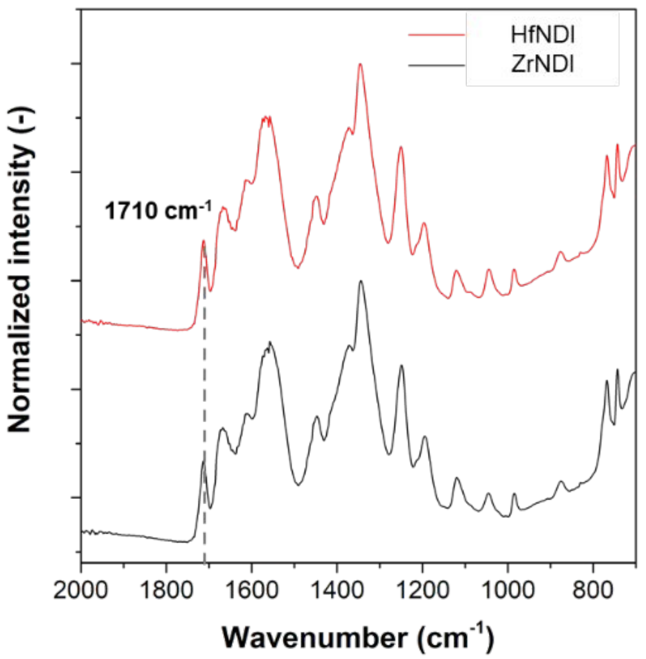

Figure S2. ATR spectrum of ZrNDI (black) and HfNDI (red). The signal at 1710 cm^{-1} indicates the residue NDI ligand trapped within the framework material.

Table S1. Cell parameters of the simulated and experimental PXRD patterns.

	**Space group**	**α (°)**	**a (Å)**	**b (Å)**	**c (Å)**	**Volume ($Å^3$)**
Simulated ZrNDI[a]	Ibam	90	16.496	25.452	36.185	15192.5
ZrNDI[b]	Ibam	90	16.849	25.162	37.487	15894.21
HfNDI[b]	Ibam	90	17.861	25.496	36.668	16698.0

[a]cell parameters are extracted from the reported cif file.[1]

[b]cell parameters are extracted from the experimental PXRD patterns in Figure 1b using GSAS II software.[2]

S2. Solid state nuclear magnetic resonance characterization of ZrNDI and HfNDI

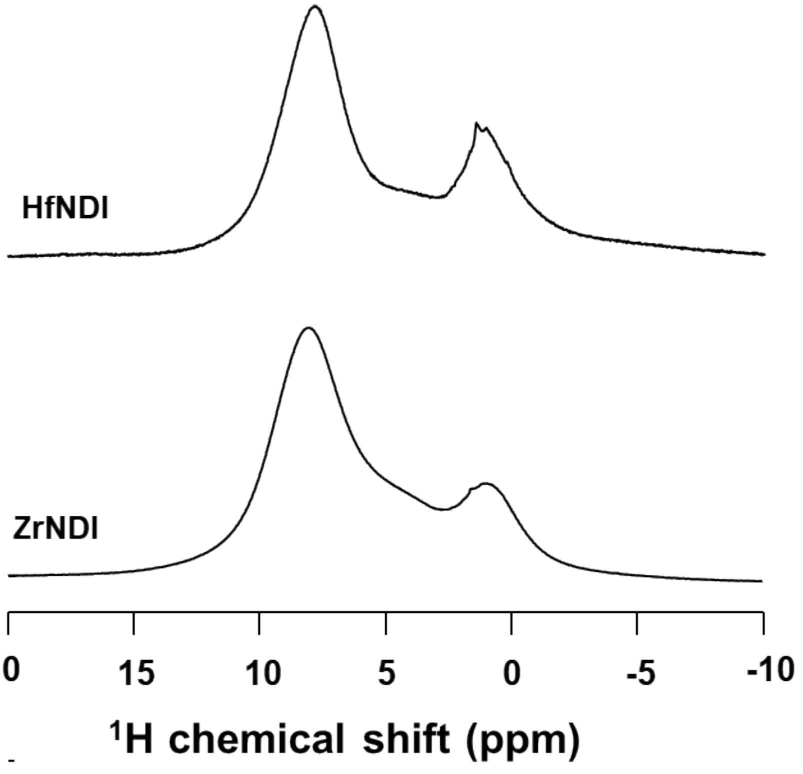

Figure S3. ^{1}H MAS NMR spectrum of trimethylphosphine oxide (TMPO) loaded ZrNDI and HfNDI.

S3. Density functional theory calculations

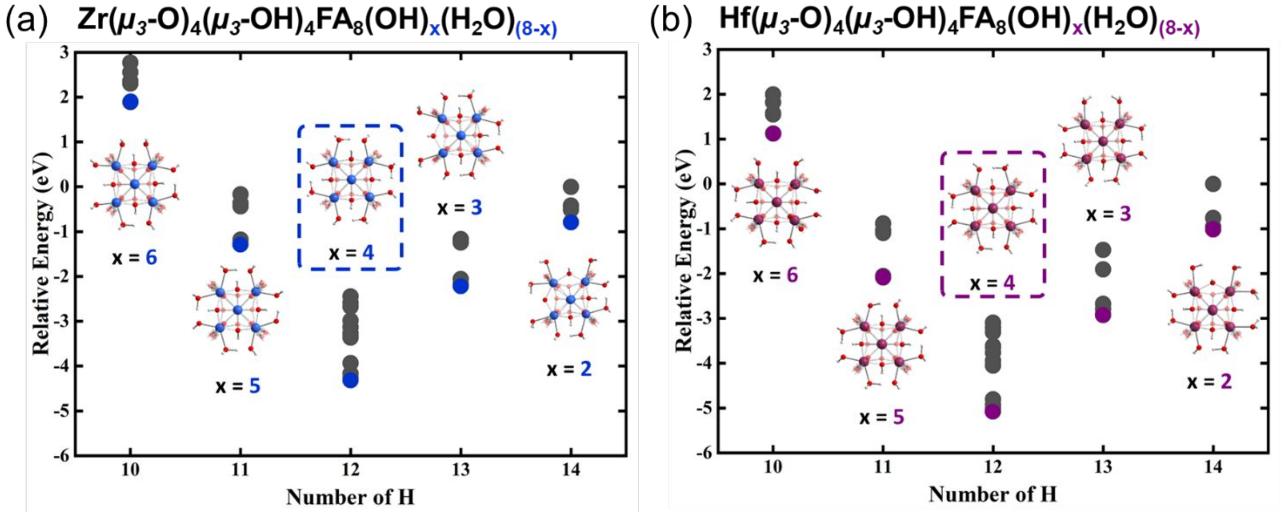

Figure S4. Coordination free energies of different number of water/hydroxyl groups at coordinatively unsaturated Zr_6 and Hf_6 oxo-cluster ($[M(\mu_3\text{-}O)_4(\mu_3\text{-}OH)_4L_8(OH)_x(H_2O)_{(8-x)}]$, M = Zr (a) or Hf (b), L = formate, x = 2, 3, 4, 5, or 6). The molecular representations of the most stable coordination configuration with a fixed quantity of water/hydroxyl groups in the Zr_6 and Hf6 oxo-cluster were also highlighted.

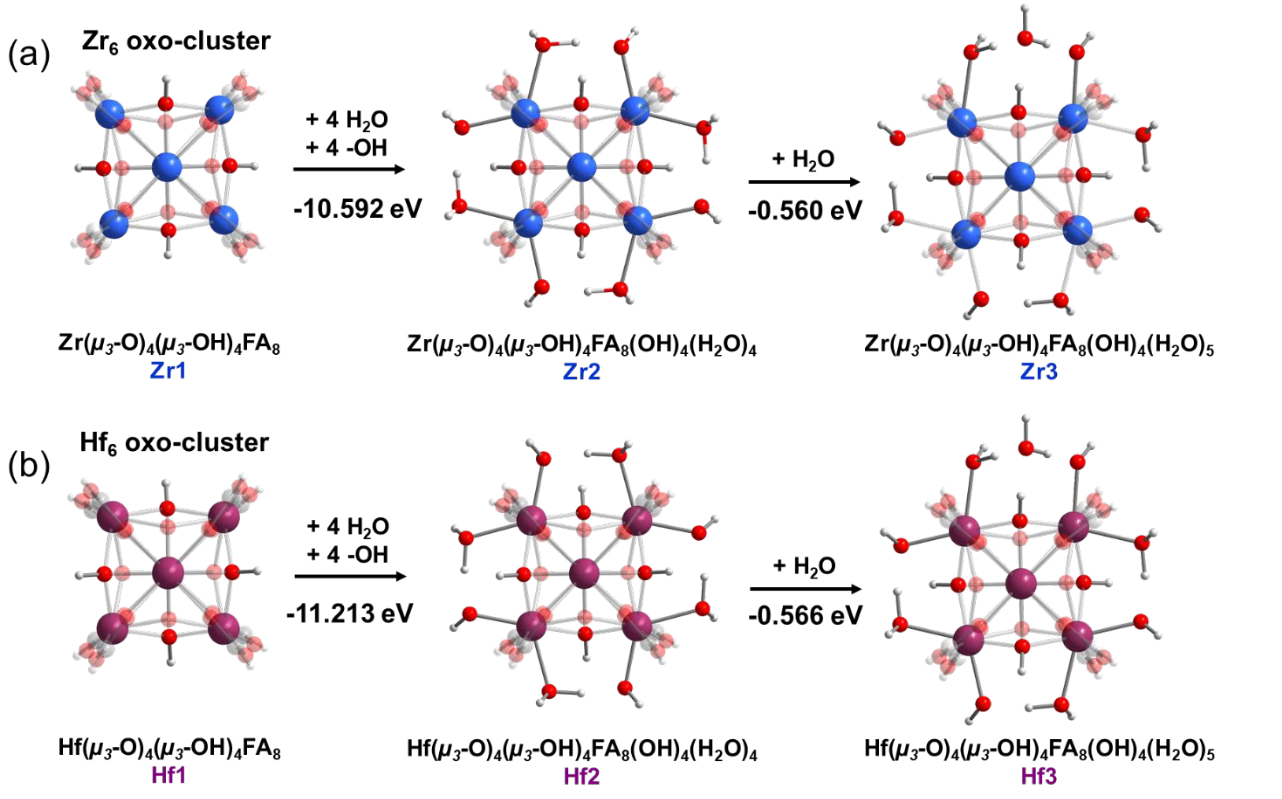

Figure S5. Molecular representations for the optimized configuration of Zr_6 oxo-cluster (a) and Hf_6 oxo-cluster (b).

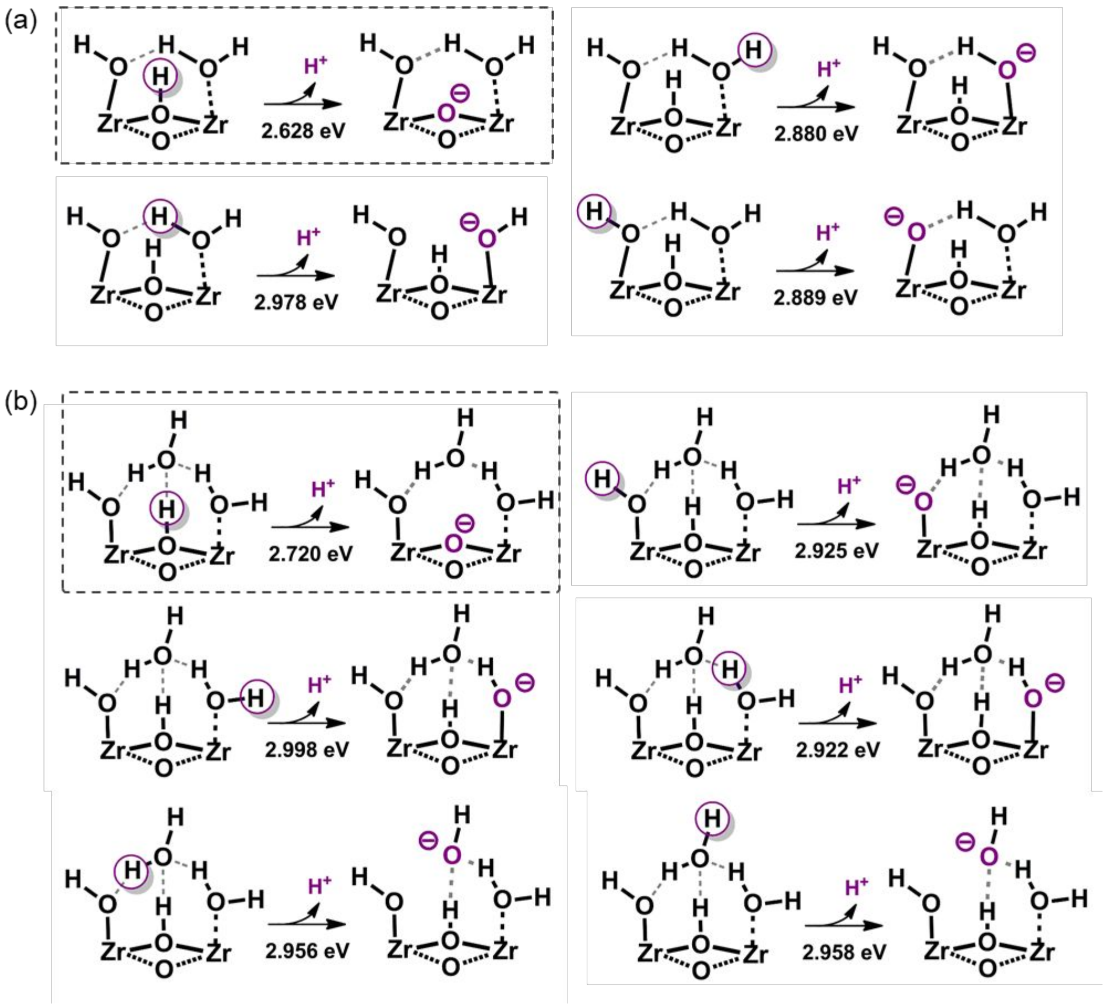

Figure S6. Schematic representations of the deprotonation pathways of Zr2 (a) and Zr3 (b). The targeted proton for deprotonation reaction in Zr2 or Zr3 cluster is highlighted in purple circle.

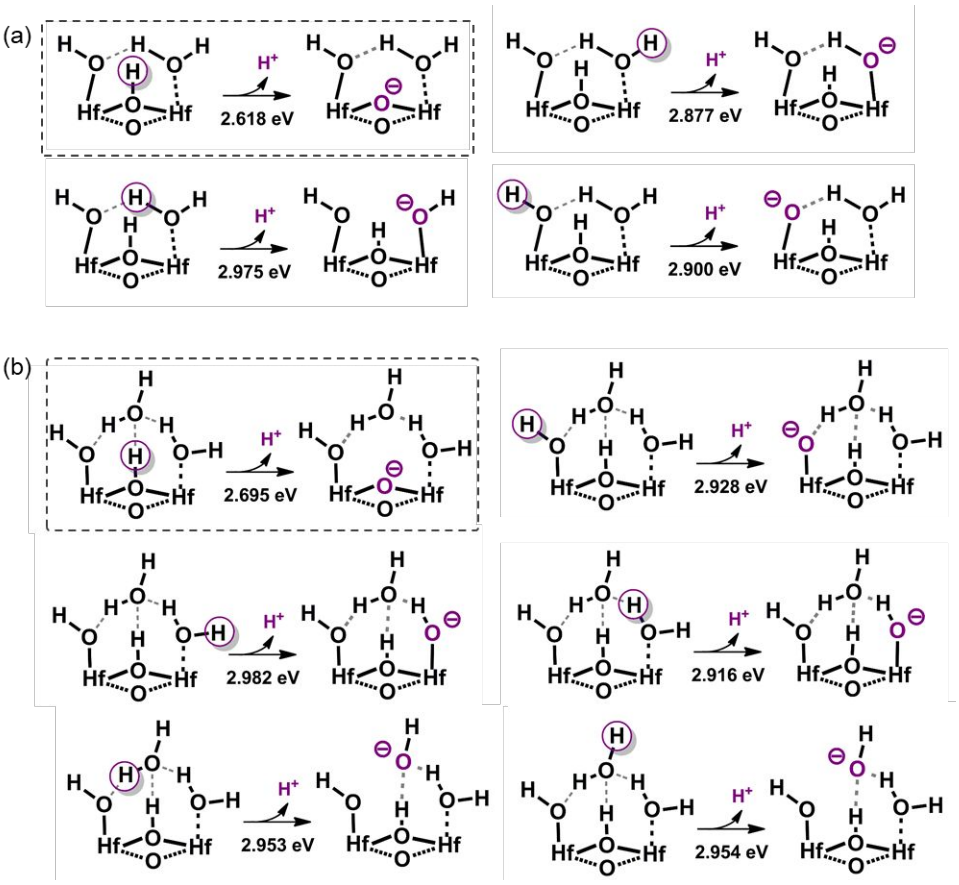

Figure S7. Schematic representations of the deprotonation pathways of Hf2 (a) and Hf3 (b). The targeted proton for deprotonation reaction in Zr2 or Zr3 cluster is highlighted in purple circle.

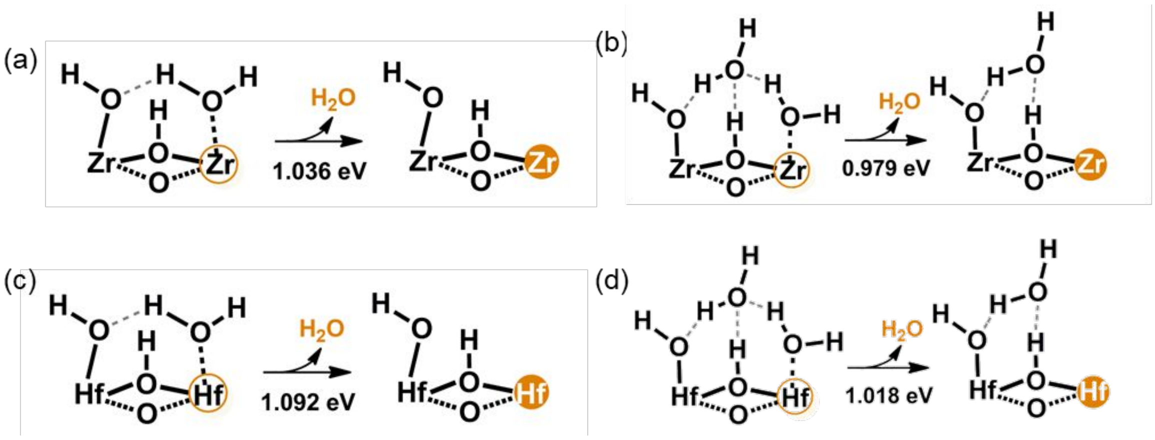

Figure S8. Schematic representations of the dehydration pathways of Zr2 (a), Zr3 (b), Hf2 (c), Hf3 (d) clusters. The resulting LAS site in the clusters are highlighted in filled orange circle.

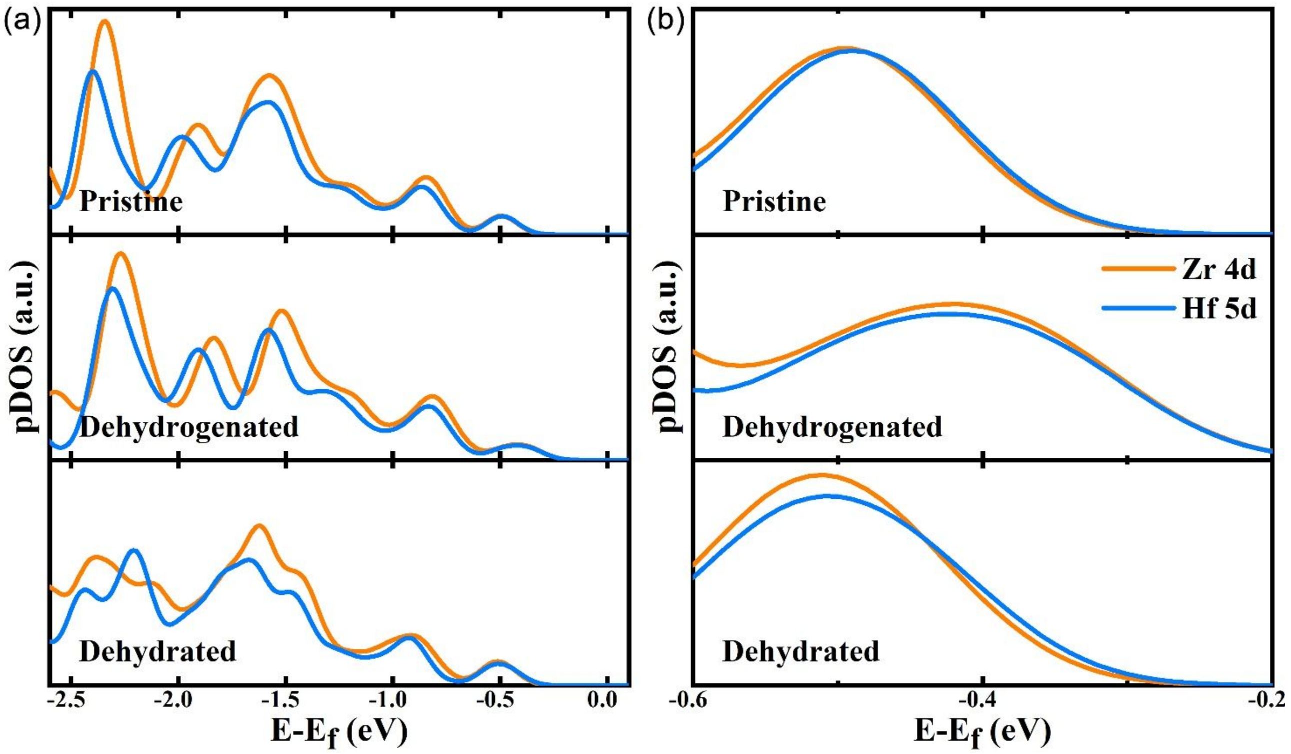

Figure S9. (a) Comparison between DFT calculated partial density of states (pDOS) of d states for Zr/Hf atom at the acid site in pristine, dehydrogenated, and dehydrated oxo-clusters. (b) zoomed in at the d band frontier near Fermi energy.

The overall partial density of d states is similar between Zr and Hf, and the band features don't change much after dehydrogenation and dehydration except for some subtle changes in the states near Fermi level. In the pristine oxo-cluster, the d states frontier of Zr cation is slightly lower in energy than that of Hf cation indicating higher stability of Zr cation. After dehydrogenation, the d states frontier of Hf cation falls behind Zr cation suggesting that Hf cation gets more stabilized than Zr cation during dehydrogenation, which explains stronger Bronsted acid site at Hf-O. In contrast, after dehydration, the energy difference between d states frontiers of Zr cation and Hf cation gets enlarged, which corresponds to the more destabilized dehydrated product of Hf oxo-cluster, and the weaker Lewis acid site at Hf.

S4. Catalytic performance of ZrNDI and HfNDI in DHA transformation reaction

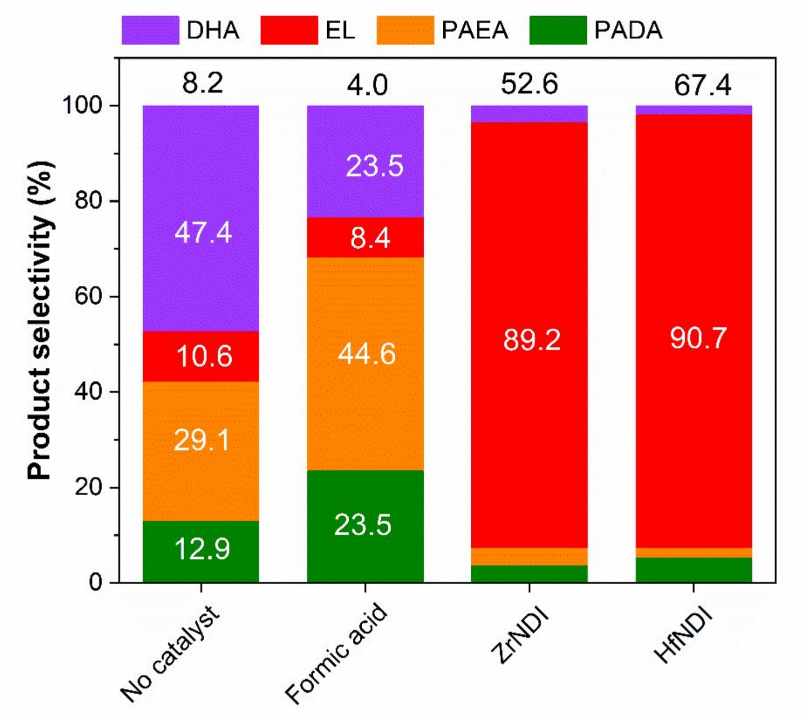

Figure S10. plots of the selectivity for PA conversion products for formic acid, ZrNDI, HfNDI, and blank test (no catalyst) after a 6h reaction in ethanol. The values of PA conversion (in percentage) of reactions are listed in black characters above each bar.

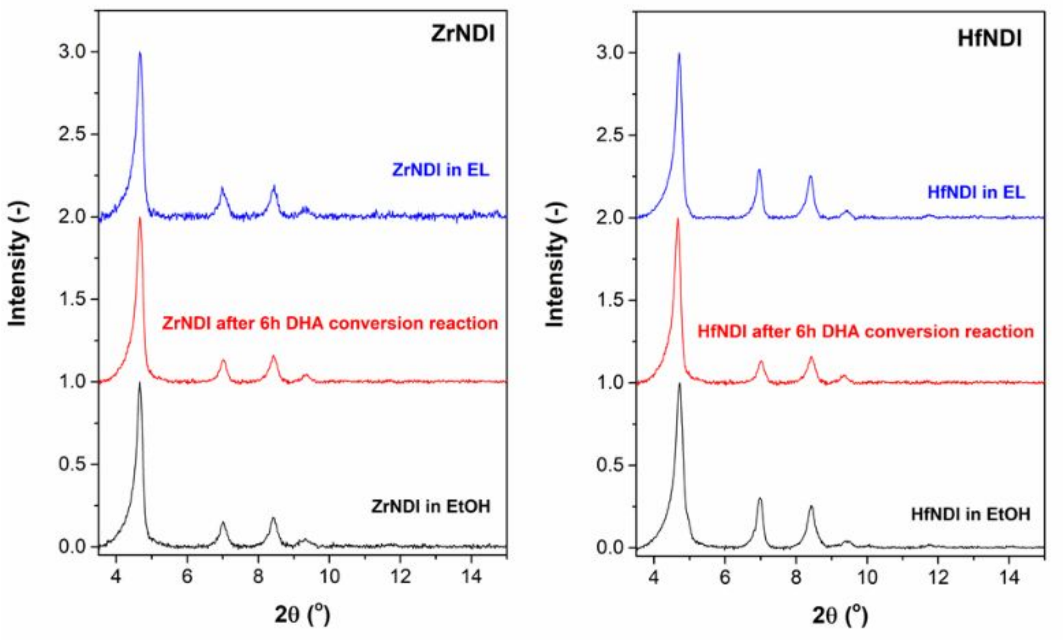

Figure S11. PXRD patterns of ZrNDI and HfNDI soaked in ethanol (black), after a 6h DHA conversion reaction (red), and soaked in ethyl lactate (blue). There is no observable shift of PXRD peaks for samples confirming the integrity as well as the absence of pore expansion/contraction in the MOF acid catalyst.

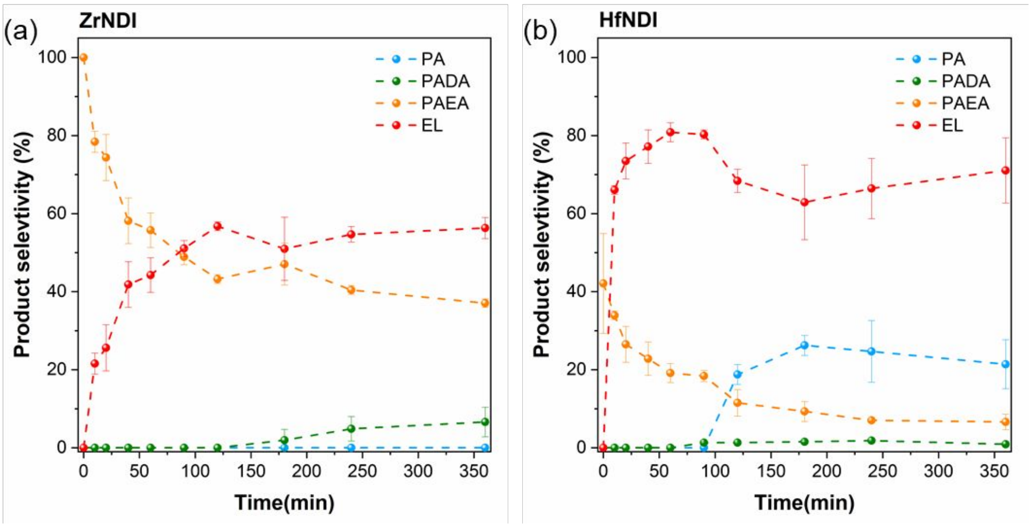

Figure S12. Integral selectivity (in percentage) of pyruvaldehyde (PA, red), pyruvaldehyde ethyl acetal (PAEA, orange), pyruvaldehyde diethyl acetal (PADA, green), and ethyl lactate (EL, red) during the catalytic DHA transformation reaction using ZrNDI (a) or HfNDI (b). Error bars in the figures represent the standard derivation calculated from triplicate experiments.

S5. Solution state NMR experiment

To further illustrate the catalytic DHA-to-EL reaction process, we performed in situ ^{1}H NMR experiments to monitor the DHA-to-EL transformation reaction over HfNDI in deuterated ethanol (d6-EtOH) at 70 °C (Figure S14a). The choice of lower reaction temperature for in situ NMR study is ascribed to the limitation of the NMR instrument in our lab. As shown in Figure S14b, after a 4h reaction at 70 °C, proton signals of the substrate (DHA) and products (PA, PAEA, and EL) are clearly visible. Based on the ^{1}H NMR data in Figure S14, the ^{1}H resonance peaks in Figure S14b at δ_{1H} = 8.03, 4.99, 4.26, 4.18, 2.13, 1.32 ppm are assigned to the aldehyde proton in PA (H_d), the -CH proton in PAEA (H_f), the aliphatic proton in DHA (H_a), the proton signals of o-carbon in EL (H_h), the proton signals in PA and PAEA (H_c and H_e), and the H_g signal in EL (H_g), respectively. It is worth ~~to~~ noting that the yields of PA, PAEA and EL were not zero at 0 min at 70 °C, due to the progression of DHA transformation reaction during the slow temperature ramping period (5 °C per 15 min, 25 °C to 70 °C) in in-situ NMR experiment. By comparing the integration areas and the difference spectra (Figure S14c-5e), we successfully calculated the kinetics parameters of each chemical in the reaction (Figure S14f, S14g, and S13). In general, the ^{1}H NMR signal related to the PA and EL products progressively increased together accompanied with a gradual depletion of DHA over the experimental period. The product signal of PAEA (-CH, δ_{1H} = 5.0 ppm) was observed to gradually decrease over the experimental period (Figure S13). As expected, no PADA side product was detected which corroborates well with the catalytic data shown in Figure 4. The selectivity of EL was then calculated to be approximately 71.4% (calculated based on the integration area in the ^{1}H NMR spectra measured after 4h reaction). This value is qualitatively consistent with the one reported in Figure 4b. The evolution rate for EL (based on δ_{1H} = 4.18 ppm) are calculated to be 2.5 times faster than PA (based on δ_{1H} = 8.03), with a value of ~0.006 min^{-1} (Figure S13). The depletion rate of DHA is approximately 2-times faster with a value of ~0.012 min^{-1}.

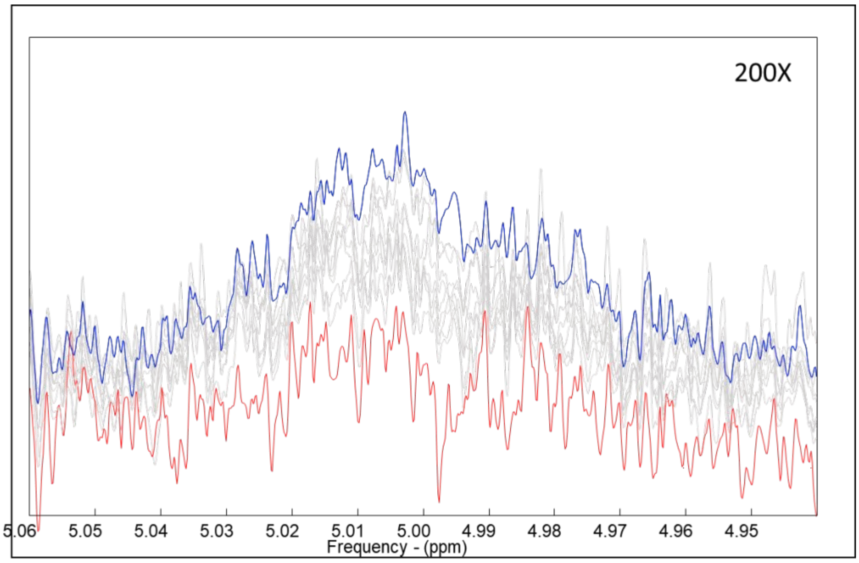

Figure S13. The expanded area of the in-situ ^{1}H NMR spectra in the region of 4.94 – 5.06 ppm for the H_f in PAEA. Blue and red lines represent the ^{1}H NMR spectra of the reaction at 0 (blue) and 4 h (red), respectively. The ^{1}H NMR spectra of the reaction mixture between 0 – 4 h are shown in grey. Due to the weak ^{1}H NMR signal, attempts to integrate these peaks for kinetics study were unsuccessful.

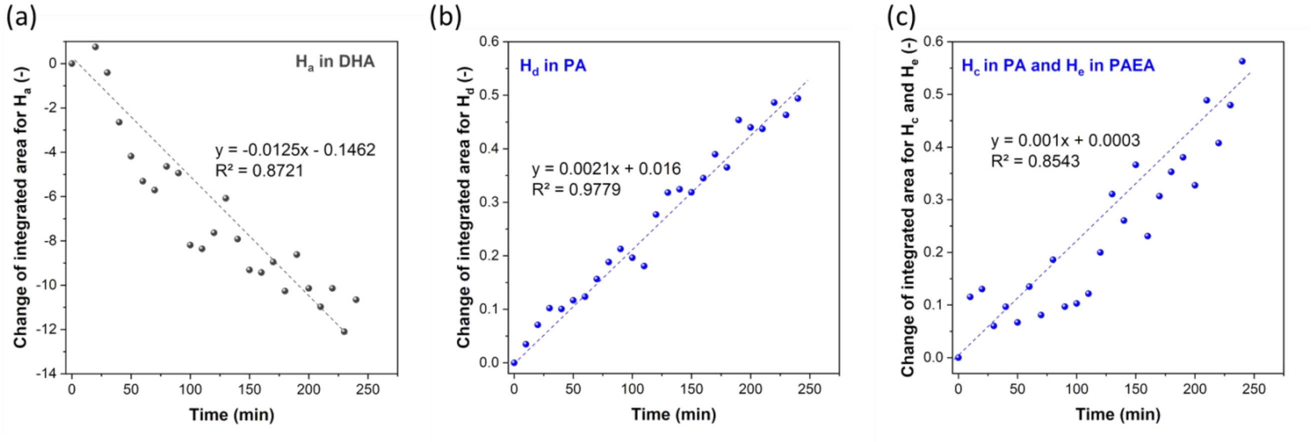

Figure S14. The progression of integrated area of H_a in DHA (a), H_d in PA (b), as well as H_c in PA and H_e in PAEA (d).

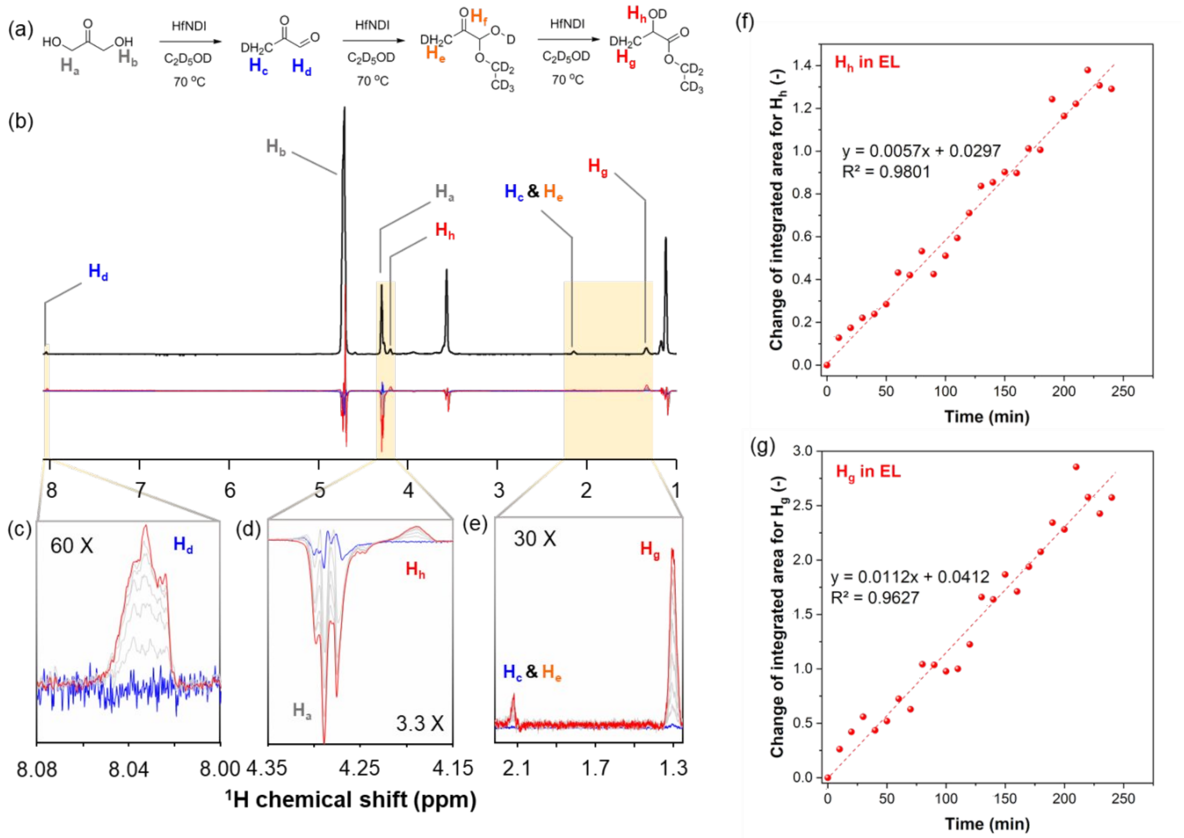

Figure S15. Monitoring the DHA-to-EL transformation reaction using HfNDI as the catalyst by ^{1}H NMR. (a) the reaction equation of the DHA-to-EL transformation reaction. (b) The ^{1}H NMR spectrum of the reaction after 4 h reaction at 70 °C (black) and the different spectra of the reaction mixture at 5 min (blue) and 4 h (red) at 70 °C. The expanded area of the difference spectra in the highlighted region for the H_d in PA (c), H_a in DHA and H_h in EL (d), as well as H_e and H_e in PA and PAEA (e), respectively, are also presented. The difference spectra of the reaction mixture between 0 – 4 h are shown in grey. The changes of integrated area for H_h (h) and H_g (g) in EL over 4 h reaction are also shown.

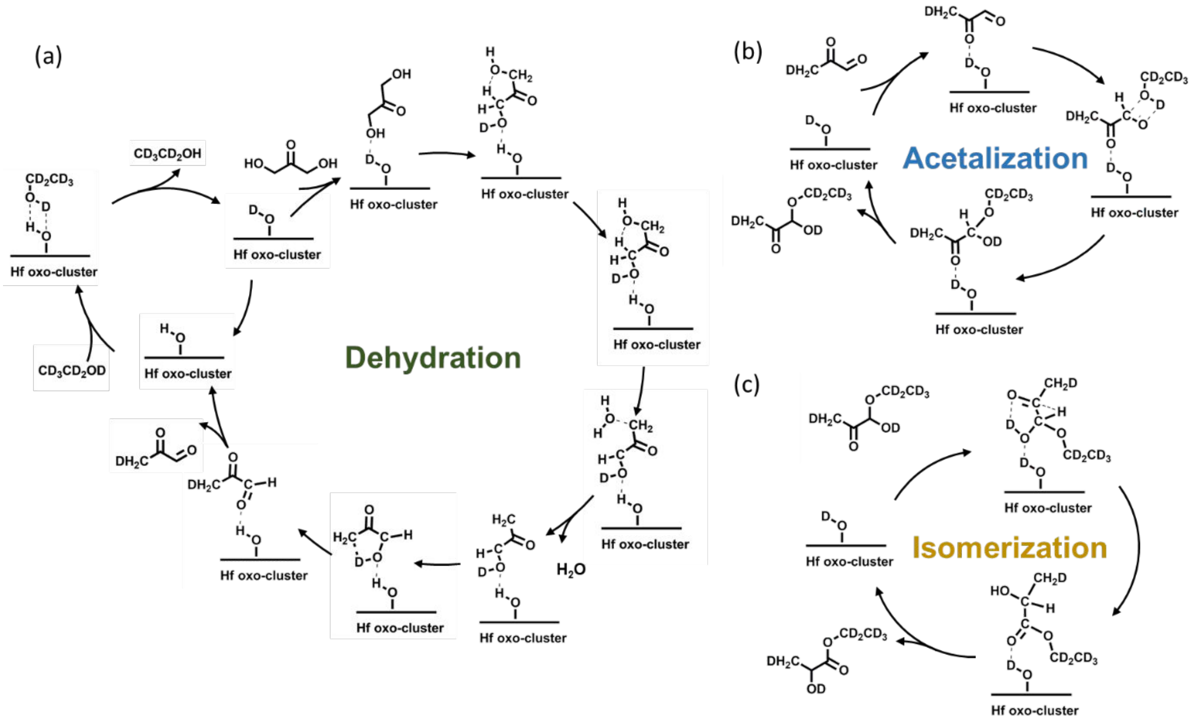

Figure S16. Proposed reaction pathway of the DHA-to-EL transformation reaction using HfNDI catalyst.

S6. Catalytic DHA-to-EL transformation reaction using Hf-MOF-808 and Hf-STA-26

To demonstrate the essential role of Hf_6 oxo-cluster in an efficient DHA-to-EL transformation reaction, we further explore the catalytic performance of another two Hf-based MOF, Hf-STA-26[3] and Hf-MOF-808.[4] Both Hf-STA-26 and Hf-MOF-808 possess intrinsic coordinatively unsaturated sites on their Hf_6 oxo-clusters. Hf-MOF-808 is constructed from six-connected Hf_6 oxo-clusters and three-connected 1,3,5-benzenetricarboxylate ligands.[4-5] Hf-STA-26 is a two-fold interpenetrated structure constructed from eight-connected Hf_6 oxo-clusters and three-connected CTTA ligand.[3, 6] Hf-MOF-808 and Hf-STA-26 possess six and eight, respectively, intrinsic coordinatively unsaturated sites on each of their structural Hf_6 oxo-cluster.[3-4]

Hf-STA-26 and Hf-MOF-808 were synthesized according to the literature using $HfCl_4$ as the metal salt and formic acid as modulator.[4, 6-8] It is worthy to note that the non-interpenetrated version of Hf-STA-26, Hf-NU-1200,[6] was reported by using benzoic acid as modulator.[3] The post-synthesis activating protocol for Hf-STA-26 and Hf-MOF-808 was analogous to that of HfNDI. PXRD measurements confirmed the successful synthesis of the targeted materials (Figure S17b and S17d). The porosity of activated Hf-STA-26 and Hf-MOF-808 was examined by using 77 K N_2 sorption analysis. The values of BET surface area were calculated to be 1094 and 1572 m^2 g^{-1} for Hf-STA-26 and Hf-MOF-808, respectively, which are consistent to the reported values (Figure S18).[4-5] The average pore sizes were determined to be 8.4 and 13.6 Å in diameter for Hf-STA-26 and Hf-MOF-808, respectively (Figure S18b); which are in line with the crystallographic data and the reported values.[3-4]

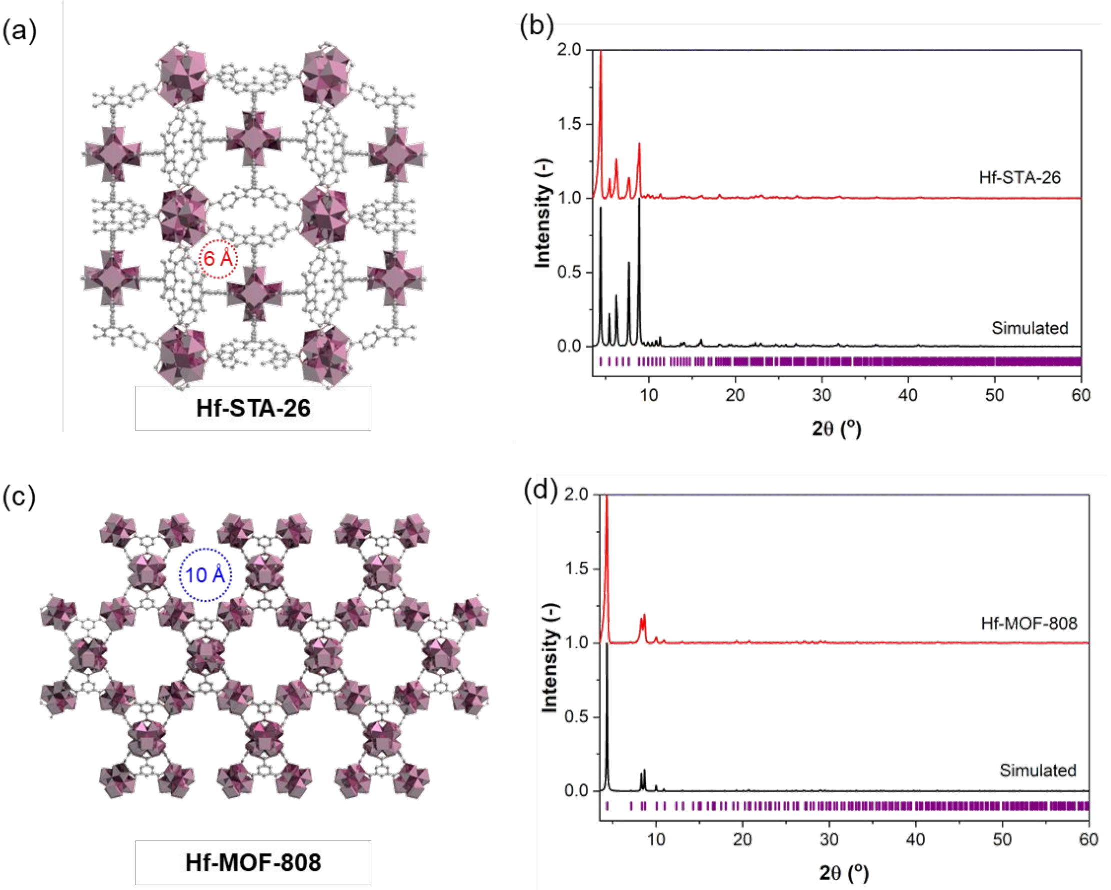

Figure S17. Molecular structure of Hf-STA-26 (a) and Hf-MOF-808 (d). The experimental and simulated PXRD pattern for Hf-STA-26 (c) and Hf-MOF-808 (d).

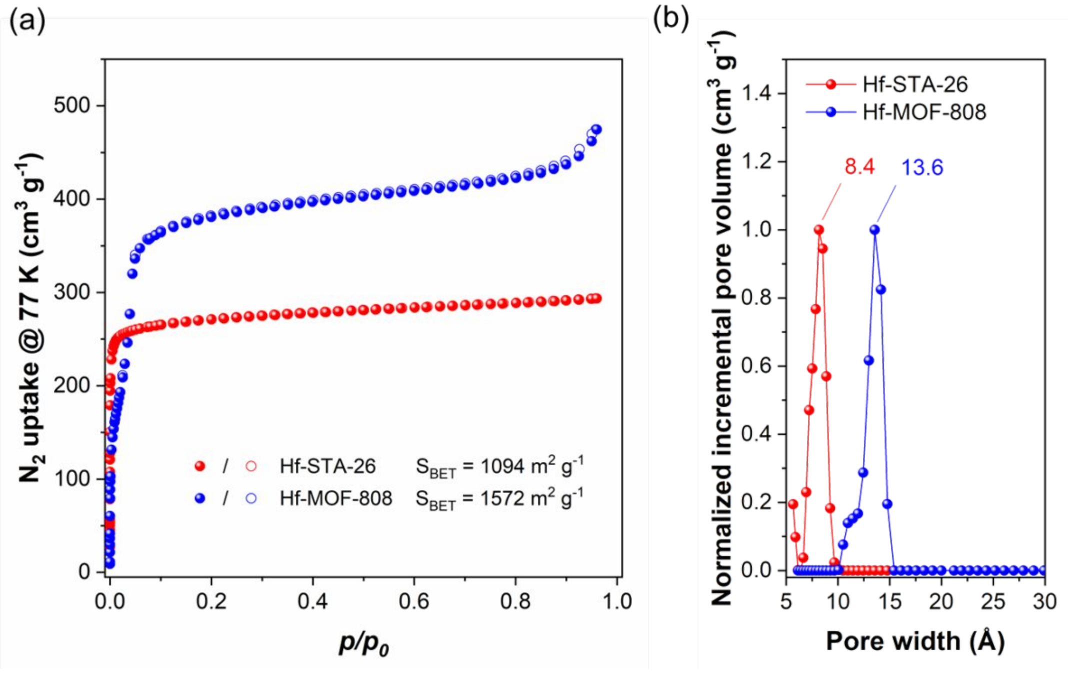

Figure S18. (c) N_2 (measured at 77 K) adsorption (filled symbols)/desorption (open symbols) isotherms for Hf-STA-26 (red) and Hf-MOF-808(blue). The calculated values of BET surface area of the materials are shown in the legend of the figure; (d) pore size distribution curves (calculated using the NLDFT model) for Hf-STA-26 (red) and Hf-MOF-808 (blue). The average pore size for Hf-STA-26 and Hf-MOF-808 were estimated at 8.4 and 13.6 Å, respectively.

With the successful synthesis of Hf-STA-26 and Hf-MOF-808, we then examined the catalytic performance of these two samples in the application of DHA-to-EL transformation reaction. The protocol of catalytic performance test is analogous to that of ZrNDI and HfNDI instead of the use of Hf-STA-26 or Hf-MOF-808 as solid acid catalyst. The catalytic results were summarized and shown in Figure S19. A noteworthy result is that Hf-STA-26 can drive a complete DHA conversion within a 6-h reaction with an 84.1% EL selectivity (Figure S19). The initial reaction for Hf-STA-26 was calculated to be 54.2 μmol g^{-1} min^{-1}, which is 7-times to that for HfNDI. The superior catalytic performance for Hf-STA-26, as compared to HfNDI, was attributed to a more opening pore structure in Hf-STA-26. The crystallographic pore aperture was estimated to be 6 Å for Hf-STA-26[3] (Figure S17) and 3 x 9 Å in HfNDI (Figure S20). A more opening pore structure in MOF was reported to be beneficial to the substrate/product diffusion, resulting in a higher reaction rate.[9] Interestingly, even with the largest pore window (ø = 10 Å[4-5], Figure S17) amongst the studied samples, Hf-MOF-808 drive the DHA conversion in a slowest rate (6.46 μmol g^{-1} min^{-1} for Hf-MOF-808 versus 8.78 μmol min^{-1} g^{-1} for HfNDI or 54.2 μmol min^{-1} g^{-1} for Hf-STA-26). Considering an envisioned better substrate/product diffusion in Hf-MOF-808, we attributed the observed slower reaction kinetics for Hf-MOF-808 to the difference of acidity composition (type/strength/density) in this material. Hf-MOF-808 is a (3,6)-connected framework with six coordinatively unsaturated sites on its Hf_6 oxo-cluster; while in HfNDI and Hf-STA-26, the number of coordinatively unsaturated sites are 8 and 4, respectively. In a potentiometric acid-base titration study, the

acidity and proton topology in Zr- or Hf-based MOFs were evaluated to be structure-dependent.[10] A systematic study is ongoing in our labs to unveil the relationship between the number/position of the coordinatively unsaturated sites and the acidity properties (composition/strength/density) on a Hf_6 oxo-cluster.

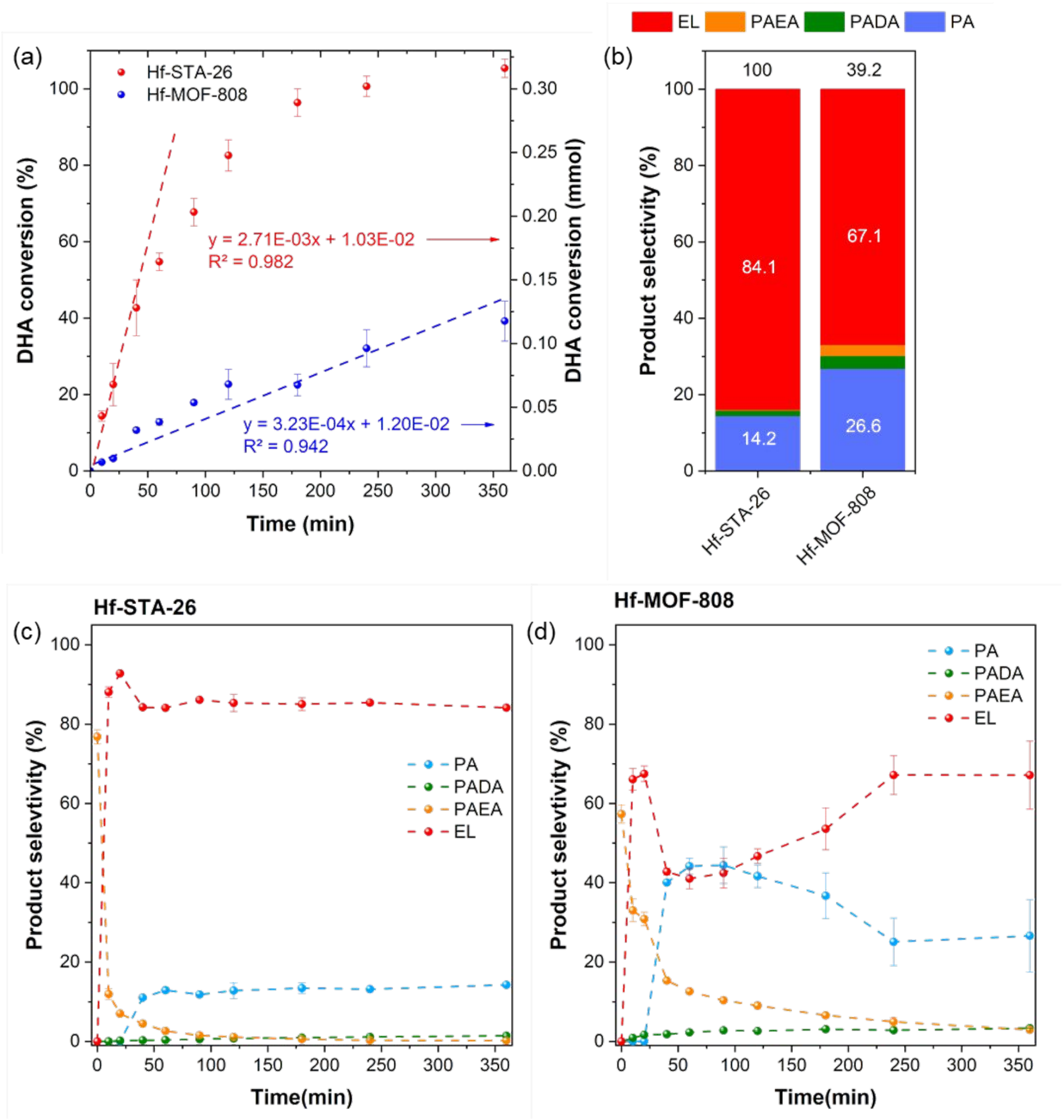

Figure S19. (a) plots of the DHA conversion (in percentage) for Hf-STA-26 (red) and Hf-MOF-808 (blue); (b) plots of the selectivity for DHA conversion product for Hf-STA-26 and Hf-MOF-808 after 6 h reaction in ethanol. The values of DHA conversion (in percentage) of reactions are listed in black characters above each bar. Integral selectivity (in percentage) of pyruvaldehyde (PA, red), pyruvaldehyde ethyl acetal (PAEA, orange), pyruvaldehyde diethyl acetal (PADA, green), and ethyl lactate (EL, red) during the catalytic DHA transformation reaction using Hf-STA-26 (c) or Hf-MOF-808 (d). Error bars in the figures represent the standard derivation calculated from triplicate experiments.

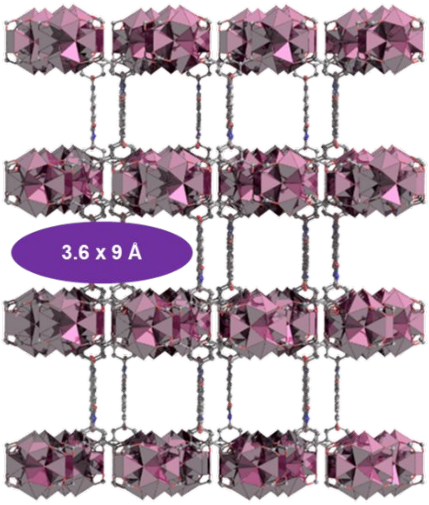

Figure S20.   Molecular representation showing the crystallographic pore opening in HfNDI.

S7. References

1. Zhang, Y.; Zhang, X.; Chen, Z.; Otake, K.-i.; Peterson, G. W.; Chen, Y.; Wang, X.; Redfern, L. R.; Goswami, S.; Li, P.; Islamoglu, T.; Wang, B.; Farha, O. K., A Flexible Interpenetrated Zirconium-Based Metal–Organic Framework with High Affinity toward Ammonia. *ChemSusChem* **2020,** *13*, 1710-1714.
2. Toby, B. H.; Von Dreele, R. B., GSAS-II: the genesis of a modern open-source all purpose crystallography software package. *J. Appl. Crystallogr.* **2013,** *46*, 544-549.
3. Bumstead, A. M.; Cordes, D. B.; Dawson, D. M.; Chakarova, K. K.; Mihaylov, M. Y.; Hobday, C. L.; Düren, T.; Hadjiivanov, K. I.; Slawin, A. M. Z.; Ashbrook, S. E.; Prasad, R. R. R.; Wright, P. A., Modulator-Controlled Synthesis of Microporous STA-26, an Interpenetrated 8,3-Connected Zirconium MOF with the the-i Topology, and its Reversible Lattice Shift. *Chem. Eur. J.* **2018,** *24*, 6115-6126.
4. Hu, Z.; Kundu, T.; Wang, Y.; Sun, Y.; Zeng, K.; Zhao, D., Modulated Hydrothermal Synthesis of Highly Stable MOF-808(Hf) for Methane Storage. *ACS Sustain. Chem. Eng.* **2020,** *8*, 17042-17053.
5. Liang, W.; Chevreau, H.; Ragon, F.; Southon, P. D.; Peterson, V. K.; D'Alessandro, D. M., Tuning pore size in a zirconium–tricarboxylate metal–organic framework. *CrystEngComm* **2014,** *16*, 6530-6533.
6. Wang, X.; Zhang, X.; Li, P.; Otake, K.-i.; Cui, Y.; Lyu, J.; Krzyaniak, M. D.; Zhang, Y.; Li, Z.; Liu, J.; Buru, C. T.; Islamoglu, T.; Wasielewski, M. R.; Li, Z.; Farha, O. K., Vanadium Catalyst on Isostructural Transition Metal, Lanthanide, and Actinide Based Metal–Organic Frameworks for Alcohol Oxidation. *J. Am. Chem. Soc.* **2019,** *141*, 8306-8314.
7. Zhao, X.; Dou, J.; Sun, D.; Cui, P.; Sun, D.; Wu, Q., A porous metal–organic framework (MOF) with unusual 2D→3D polycatenation based on honeycomb layers. *Dalton Trans.* **2012,** *41*, 1928-1930.
8. Wang, B.; Lv, X.-L.; Feng, D.; Xie, L.-H.; Zhang, J.; Li, M.; Xie, Y.; Li, J.-R.; Zhou, H.-C., Highly Stable Zr(IV)-Based Metal–Organic Frameworks for the Detection and Removal of Antibiotics and Organic Explosives in Water. *J. Am. Chem. Soc.* **2016,** *138*, 6204-6216.
9. Ma, L.; Falkowski, J. M.; Abney, C.; Lin, W., A series of isoreticular chiral metal–organic frameworks as a tunable platform for asymmetric catalysis. *Nat. Chem.* **2010,** *2*, 838-846.
10. Klet, R. C.; Liu, Y.; Wang, T. C.; Hupp, J. T.; Farha, O. K., Evaluation of Brønsted acidity and proton topology in Zr- and Hf-based metal–organic frameworks using potentiometric acid–base titration. *Journal of Materials Chemistry A* **2016,** *4*, 1479-1485.
11. de Saint Laumer, J.-Y.; Cicchetti, E.; Merle, P.; Egger, J.; Chaintreau, A., Quantification in Gas Chromatography: Prediction of Flame Ionization Detector Response Factors from Combustion Enthalpies and Molecular Structures. *Analytical Chemistry* **2010,** *82*, 6457-6462.
